# Gene signatures of circulating breast cancer cell models are a source of novel molecular determinants of metastasis and improve circulating tumor cell detection in patients

**DOI:** 10.1186/s13046-022-02259-8

**Published:** 2022-02-25

**Authors:** Emanuela Fina, Loredana Cleris, Matteo Dugo, Mara Lecchi, Chiara Maura Ciniselli, Daniele Lecis, Giulia Valeria Bianchi, Paolo Verderio, Maria Grazia Daidone, Vera Cappelletti

**Affiliations:** 1 Biomarkers Unit, Department of Applied Research and Technological Development, Fondazione IRCCS Istituto Nazionale dei Tumori di Milano, Via Venezian 1, 20133 Milan, Italy; 2 Current affiliation: Humanitas Research Center, IRCCS Humanitas Research Hospital, via Manzoni 56, Rozzano, 20089 Milan, Italy; 3 Platform of Integrated Biology, Department of Applied Research and Technological Development, Fondazione IRCCS Istituto Nazionale dei Tumori, Via Venezian 1, 20133 Milan, Italy; 4 Current affiliation: Department of Medical Oncology, IRCCS Ospedale San Raffaele, via Olgettina 60, 20132 Milan, Italy; 5 Bioinformatics and Biostatistics Unit, Fondazione IRCCS Istituto Nazionale dei Tumori, Via Venezian 1, 20133 Milan, Italy; 6 Molecular Immunology Unit, Department of Research, Fondazione IRCCS Istituto Nazionale dei Tumori, Via Venezian 1, 20133 Milan, Italy; 7 Medical Oncology Unit, Fondazione IRCCS Istituto Nazionale dei Tumori, Via Venezian 1, 20133 Milan, Italy

**Keywords:** Circulating tumor cells, Gene signatures, Metastasis, Biomarkers, Breast cancer

## Abstract

**Background:**

Progression to stage IV disease remains the main cause of breast cancer-related deaths. Increasing knowledge on the hematogenous phase of metastasis is key for exploiting the entire window of opportunity to interfere with early dissemination and to achieve a more effective disease control. Recent evidence suggests that circulating tumor cells (CTCs) possess diverse adaptive mechanisms to survive in blood and eventually metastasize, encouraging research into CTC-directed therapies.

**Methods:**

On the hypothesis that the distinguishing molecular features of CTCs reveal useful information on metastasis biology and disease outcome, we compared the transcriptome of CTCs, primary tumors, lymph-node and lung metastases of the MDA-MB-231 xenograft model, and assessed the biological role of a panel of selected genes, by in vitro and in vivo functional assays, and their clinical significance in M0 and M+ breast cancer patients.

**Results:**

We found that hematogenous dissemination is governed by a transcriptional program and identified a CTC signature that includes 192 up-regulated genes, mainly related to cell plasticity and adaptation, and 282 down-regulated genes, involved in chromatin remodeling and transcription. Among genes up-regulated in CTCs, *FADS3* was found to increases cell membrane fluidity and promote hematogenous diffusion and lung metastasis formation. *TFF3* was observed to be associated with a subset of CTCs with epithelial-like features in the experimental model and in a cohort of 44 breast cancer patients, and to play a role in cell migration, invasion and blood-borne dissemination. The analysis of clinical samples with a panel of CTC-specific genes (*ADPRHL1*, *ELF3*, *FCF1*, *TFF1* and *TFF3)* considerably improved CTC detection as compared with epithelial and tumor-associated markers both in M0 and stage IV patients, and CTC kinetics informed disease relapse in the neoadjuvant setting.

**Conclusions:**

Our findings provide evidence on the potential of a CTC-specific molecular profile as source of metastasis-relevant genes in breast cancer experimental models and in patients. Thanks to transcriptome analysis we generated a novel CTC signature in the MDA-MB-231 xenograft model, adding a new piece to the current knowledge on the key players that orchestrate tumor cell hematogenous dissemination and breast cancer metastasis, and expanding the list of CTC-related biomarkers for future validation studies.

**Supplementary Information:**

The online version contains supplementary material available at 10.1186/s13046-022-02259-8.

## Background

Hematogenous spread of cancer cells to distant organs and their growth to overt metastases are responsible for the majority of breast cancer-related deaths [1, 2]. Indeed, the 5-year survival rate drops from 99 and 86% respectively for localized and regional diseases, to 27% when cancer progresses to stage IV [3, 4], a fatal condition which includes up to 10% of cases at first diagnosis and about 30% emerging during treatment or years after surgical resection with curative intent [5]. Although longstanding breast cancer markers still play a major role in patient selection for standard treatments, and we are assisting substantial improvements in therapeutic protocols to target the primary tumor, therapies intended to prevent distant relapse or directed against an overgrowing population of metastatic cells still fail in ensuring prolonged clinical benefit [6, 7]. In recent years, trials with single immunotherapeutic agents paved the way for new alternative treatments also in breast cancer, but their success rate in the metastatic setting remains around 10% [8].

A major obstacle towards an effective treatment of advanced or stage IV breast cancer is the lack of comprehensive knowledge of the molecular mechanisms that metastasis-initiating or persistent cells activate to escape primary sites and therapies. Tumor progression typically follows a sequence of steps [9], each governed by specific genes that might represent possible targets for acting to stop the cascade definitively. However, current treatment protocols are not based on the features of disseminated cells and have shown limited chance to control metastasis in the long term. Thus, the identification of biomarkers associated with such targets is of critical importance for better risk assessment and treatment choice.

Recent technological advances in the detection and analysis of circulating tumor cells (CTCs) have fostered basic and translational research to understand their biology and search for novel cancer biomarkers [10, 11]. In addition to the leading application of CTC enumeration as complementary biomarker for disease staging [12, 13], longitudinal monitoring of disease status and design of personalized treatments [14], CTCs are considered the seeds of metastasis [15] and their crucial role as a novel experimental model to investigate cancer cell systemic spread has been shown by an increasing number of studies [16, 17]. Accordingly, analyzing the CTC transcriptome may help to elucidate the mechanisms of metastasis formation and to identify biologically relevant CTC-related genes as alternative non-invasive biomarkers of clinical interest. Still, further investigation is needed to capture the full message on the dissemination process by CTCs and to translate it to the clinics.

To reduce this knowledge gap, we designed a study on the hypothesis that hematogenous dissemination is a step of the metastatic cascade whose effectiveness is orchestrated by a specific group of genes. We performed gene expression profiling experiments to characterize CTCs, and compared their transcriptome with those of primary and secondary solid lesions obtained from orthotopic xenograft models of the MDA-MB-231 breast cancer cell line. The significance of a panel of genes differentially expressed in CTCs was investigated by in vitro and in vivo functional assays and by transcript analysis of CTC-enriched blood samples from cohorts of early (M0) and advanced (M+) breast cancer patients.

## Methods

Circulating tumor cell analysis was performed in the MDA-MB-231 (ATCC; Manassas, VA, USA) orthotopic xenograft model (female NOD SCID mice, Charles River, Wilmington, MA, USA) [18, 19] and in breast cancer patients candidate to multimodal treatment (*N*=20) or to first-line systemic therapy for metastatic disease (*N*=31), before starting therapy and during the course of treatment whenever possible. CTCs were isolated by immunoaffinity-based (AdnaTest^TM^, AdnaGen AG, Langenhagen, Germany) [20] and size-based filtration methods (ScreenCell® kits, ScreenCell, Sarcelles, France) [18], and enumerated by indirect quantification through a PCR-based approach or by direct count on pre-stained filters. Gene expression profiles were obtained by the Whole-Genome DASL® HT assay (Illumina, Inc., San Diego, CA, USA) [21] in xenograft models and by low-density array for a selected panel of genes (Custom TaqMan® Gene Expression Array Cards, Thermo Fisher Scientific, Waltham, MA, USA) in patients [22]. Gene expression profiles of the MDA-MB-231 cell line and of tissues obtained from xenograft models at animal sacrifice (CTC-enriched blood sample, and sections of primary tumor, lymph-nodes and lungs) were analyzed by using R/Bioconductor “lumi” [23, 24] and the “topGO” Gene Ontology packages. Statistical analysis was carried out with SAS (Statistical Analysis System, RRID:SCR_008567, version 9.4; SAS Institute, Inc., Cary, NC, USA) adopting an α level of 5%. Graphical representations were obtained with Prism version 9.2 (GraphPad Software, San Diego, CA, USA). Data of in vitro functional assays are the result of experiments run in technical triplicate on three batches of cells separately infected with shRNA-lentiviral particles. Each lentiviral infection replicate is labelled in graph with a distinct color, each dot represents one well. Data from each animal is labelled in dot plot with a distinct color. Single CTC, CTC cluster and metastasis counts in the same animal were labelled with the same color. Clinical and animal studies were approved by the Ethics Committees at Fondazione IRCCS Istituto Nazionale dei Tumori (Milan, Italy). Detailed information on the methodology is available in the Supplementary information files.

## Results

### Species-specific assays enable the quantification and gene expression profiling of CTCs in the mouse background of MDA-MB-231 xenograft models

**Fig. 1:**
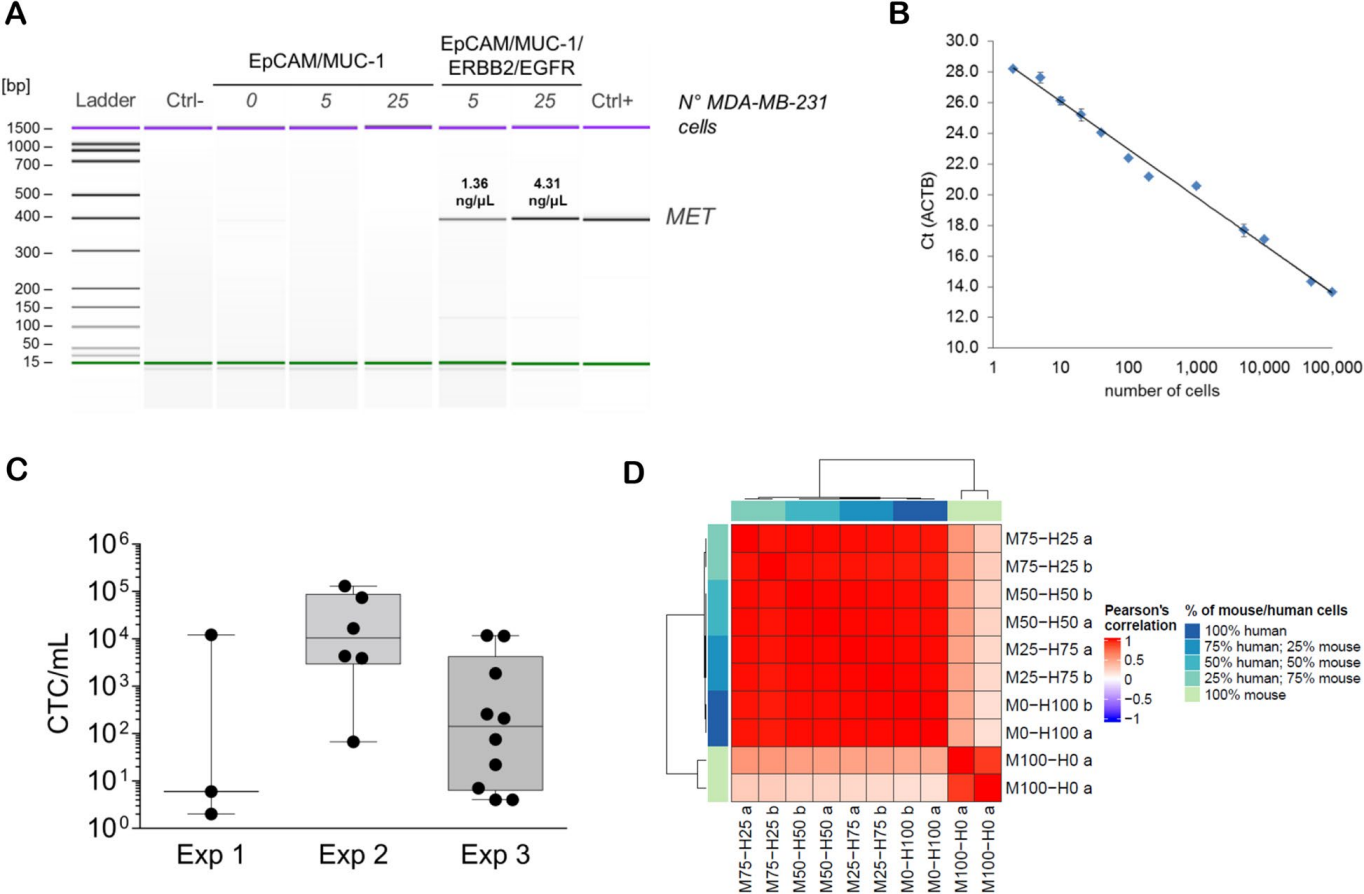
Circulating tumor cell (CTC) enrichment and gene expression profile (GEP) analysis in MDA-MB-231 xenograft models. **A** Gel-like image of a capillary electrophoresis separation of *MET* amplicon following MDA-MB-231 cell spike-in and immuno-capture experiments. **B** Logarithmic curve of mean±standard deviation threshold cycles obtained by quantitative PCR for human *ACTB* as a function of MDA-MB-231 cell numbers (*n*=3 experimental replicates per point). **C** Dot plot of CTC numbers per blood volume in MDA-MB-231 xenograft models (*n*=3 experiments). **D** Heat map of pair-wise correlations using raw GEP data from technical duplicates (“a” and “b”) of human (“H”) and murine (“M”) universal reference RNA inputs mixed at different ratios (numbers indicate the RNA percentage)

We first developed technical protocols applicable to MDA-MB-231 xenograft models for CTC detection and gene expression profile analysis. To optimize the enrichment for CTCs with down-regulated expression of epithelial markers we used magnetic particles coated with a cocktail of antibodies directed against HER2 and EGFR in addition to EpCAM and MUC1. The capture efficiency was tested in spike-in experiments by assessing the amplification level of *MET* gene, which was detectable in blood samples spiked-in with as few as 5 MDA-MB-231 cells and showed increased signal intensity in samples spiked-in with 25 cells (Fig. 1A). In order to quantify human tumor cells in specimens of xenograft models for gene expression profile experiments, we constructed a standard curve by plotting threshold cycles obtained by a human β-actin-specific qPCR assay as a function of MDA-MB-231 cell numbers (Fig. 1B). We next estimated the variability of the CTC load in the MDA-MB-231 xenograft model, starting from a pilot experiment on three animals to test our CTC capture and quantification method, and then increasing the group size in order to assess the variability in CTC frequency (Fig. 1C). The tumor and metastasis take rates were 100%. However, although CTCs could be found in all cases, the number of cells was variable (overall median (Interquartile Range, IQR) CTC number per milliliter of blood: 258 (7-11,784)) according to the indirect quantification protocol. We finally run a preliminary test to assess the species-specificity of the microarray probes by using human and murine universal RNA reference samples mixed at different ratios (25-50-75%) in order to simulate biological samples derived from xenograft models. The distribution of signal intensities was comparable in all samples containing human RNA, regardless of their percentage (Supplementary Fig. S1, left), while the detection rates and mean signal intensities were negligible in pure murine RNA samples (Supplementary Fig. S1, right), which clustered separately from the others (Fig. 1D), thus proving that the platform is specific for the human transcriptome and suitable to gene expression experiments with xenograft models.

### A transcriptional reprogramming starts the hematogenous phase of breast cancer metastasis

We ran two independent experiments (hereafter referred to as “GEP1” and “GEP2”) using groups of three animals each with an overall median (IQR) total number of 28,121 (13,577-42,619) CTCs (Supplementary Table S1). Gene expression data were obtained for CTCs, cells disseminated to the bone marrow (DTC), primary tumor nodules (PT), lymph-nodal (LN) and pulmonary metastases (LUNG), and for cultures of the parental MDA-MB-231 cell line. Quality control tests showed raw log_2_(signal intensities) ranging from 6 to 10 and probe detection rates around 60% (Supplementary Fig. S2, panel A). Reciprocal correlation analyses revealed one main cluster and four scarcely correlated samples, one each in animals 70X and 152X, and the other two in animal 147X (Supplementary Fig. S2, panel B). The distribution of normalized signal intensities appeared homogenous among all samples retained after excluding low-performance samples (Supplementary Fig. S2, panel C), thus validating the robustness of our technical protocol for tumor cell quantification and gene profiling in xenograft models.

**Fig. 2:**
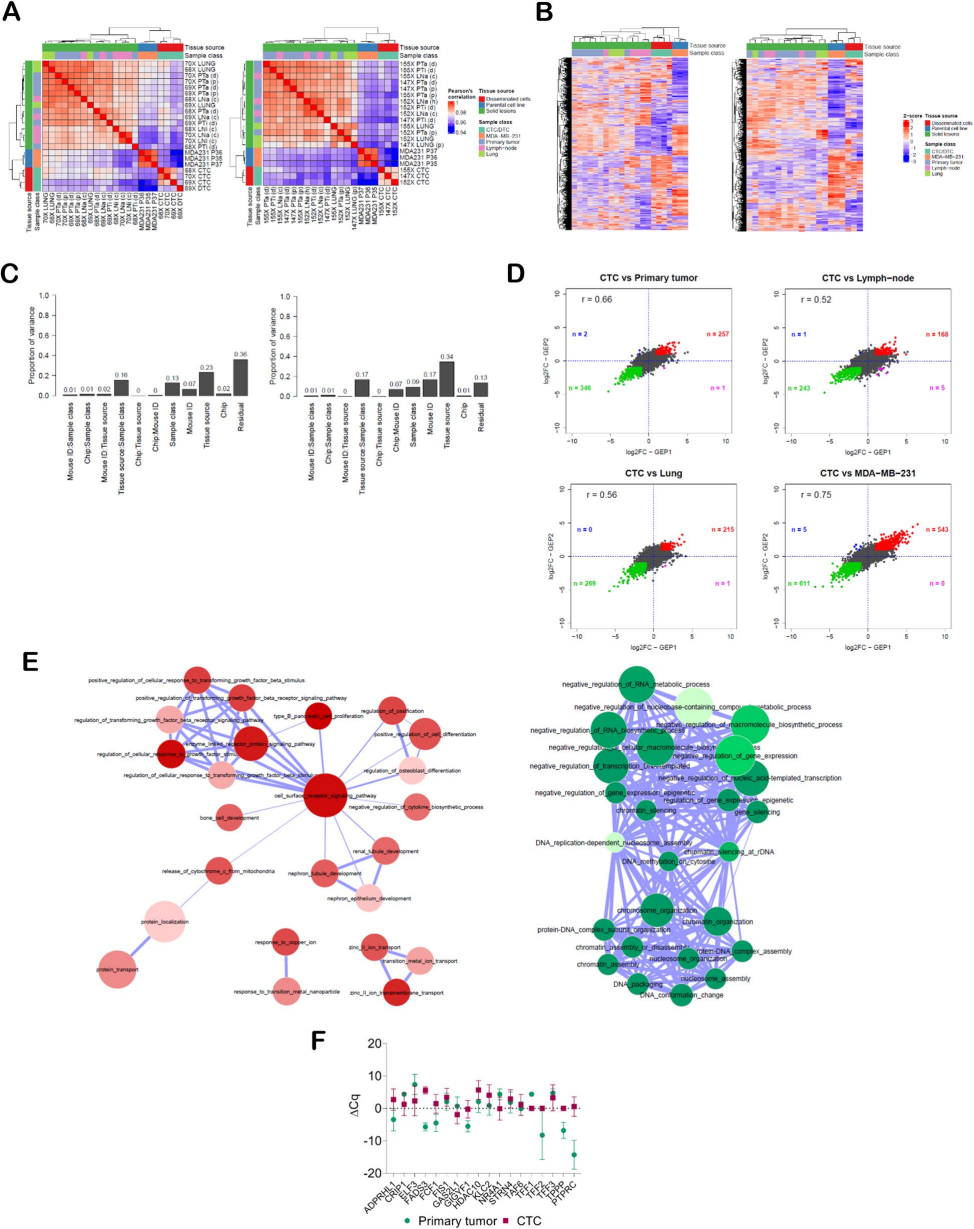
Circulating tumor cells (CTCs) undergo massive transcriptional reprogramming and possess a distinct gene expression profile (GEP). **A** Heat map representation of pair-wise correlations of normalized expression data from (left) GEP1 and (right) GEP2 experiments in MDA-MB-231 xenografts (“a”, axillary; “i”, inguinal; “(c)”, controlateral to PT; “(h)”, homolateral to PT; “(d)”, distal section; “(p)”, proximal section). **B** Heat map representation of the expression pattern of the most variable genes (interquartile range intensities >95^th^ percentile) in (left) GEP1 and (right) GEP2. **C** Bar chart of the experimental variability according to principal variance component analysis in (left) GEP1 and (right) GEP2. **D** Scatter plot of correlations among log_2_(fold changes) considering all detected genes (dots) in GEP1 and GEP2, in pair-wise sample class comparisons. Genes significantly up-regulated or down-regulated (fold change threshold=│2│and false discovery rate <0.05) in both experiments are represented by red and green dots, respectively, and genes with discordant trend are represented by pink or blue dots if up-regulated in GEP1 AND down-regulated in GEP2 or down-regulated in GEP1 AND up-regulated in GEP2, respectively. **E** Network representation of Gene Ontology (GO) terms enriched in the lists of genes significantly (left) up-regulated or (right) down-regulated in the comparison between CTCs and solid lesions using GEP1 and GEP2 data. Nodes represent significantly enriched GO terms, size and color are proportional to the number of genes annotated in the term and to their significance (darker color, higher statistical significance), respectively. Nodes that share common genes are connected by an edge, with thickness proportional to the overlap coefficient (OC) between the two terms, calculated as |A∩B|/min(|A|, |B|). Only terms with an OC≥0.5 are shown. **F** Plot of the mean±standard deviation relative expression (ΔC_q_, normalized equivalent threshold cycle) of a panel of MDA-MB-231-CTC up-regulated genes and of CD45 gene (*PTPRC*) in the CTC fraction and tumor biopsy of *n*=4 patients

Reciprocal correlation analysis of normalized data highlighted three main clusters: the group of CTC and DTC samples, a larger group including solid tumor lesions (PT, LN and LUNG sample classes), and the parental cell line (Fig. 2A). No sub-clusters of samples belonging to the same class emerged within the principal cluster of solid tumor lesions. Unsupervised hierarchical clustering using the most variable genes (*n*=209, IQR intensities >95^th^ percentile) provided evidence that, despite the common origin, MDA-MB-231 cells, tumor lesions and CTCs/DTCs do possess distinct transcriptome profiles (Fig. 2B). Indeed, when looking at the cluster of highly expressed genes, MDA-MB-231 cells shared about 50% of such genes with CTCs, and CTCs in turn were exclusively characterized by a fraction of genes (roughly 70% in GEP1, and ranging from 20 to 50%, based on the CTC sample, in GEP2) showing different expression levels compared to the parental cell line. Moreover, the overlap between genes highly expressed in CTCs and in solid tumor lesions was slight, again supporting the existence of unique CTC molecular traits (Fig. 2B). Principal variance component analysis confirmed that the differences observed in the CTC transcriptome compared to the other lesions mirror their biological features and are not the result of an experimental artifact, as the tissue source (i.e., disseminated cells, parental cells and solid lesions), which accounted for 23% in GEP1 and 34% in GEP2, was the factor that mostly contributed to the overall variability compared to the sample class or other experimental and technical factors (Fig. 2C). Finally, gene expression data in GEP1 were consistent with those observed in GEP2 as correlation values between fold changes (FC) obtained in both experiments for all detected genes were higher than 0.5 in all pair-wise class comparisons between CTC and PT, LN, LUNG or MDA-MB-231 samples (Fig. 2D).

With the aim to investigate the biological meaning of gene expression data, we first listed genes significantly modulated (FC threshold=│2│and false discovery rate (FDR) <0.05) in MDA-MB-231 CTCs compared to all solid lesions in GEP1 and GEP2, i.e., tissue sections of primary tumor nodules, lymph nodes and lungs, corresponding to *n*=15 samples in GEP1 and *n*=14 samples in GEP2, and found that those common to both experiments actually accounted for a total of 192 up-regulated and 282 down-regulated genes (Supplementary file S1). Up-regulated genes were enriched in Gene Ontology (GO) terms related to embryogenesis, development and morphogenesis of various tissues and organs, especially bone, neural, renal and vascular systems, as also cell adhesion, motility, metabolism, and response to physical, chemical and biological external stimuli (Fig. 2E, left; Supplementary file S2), suggesting a remarkable CTC plasticity and adaptation ability. Instead, down-regulated genes were enriched in GO terms mainly related to chromatin remodeling and negative regulation of transcription, which is consistent with the strong gene modulation observed in CTCs compared to solid lesions (Fig. 2E, right; Supplementary file S3).

We tested in tissues derived from breast cancer patients (2 M0 and 2 with subsequent diagnosis of distant metastases) the expression level of a panel of 17 genes selected among those up-regulated in experimentally-derived CTCs by comparing CTC-enriched blood samples collected at baseline with fine-needle biopsies obtained from matched primary tumors. *ADPRHL1*, *FADS3*, *FCF1*, *FIS1*, *GIGYF1*, *HDAC10*, *KLC2*, *STRN4* and *TAF6* were expressed at least in one CTC sample and showed a trend toward higher expression values in the CTC population compared to cancer cells at primary site (Fig. 2F; plot of individual gene expression data in Supplementary Fig. S2, panel D), thus supporting findings arising from gene expression analysis of CTC models obtained in the MDA-MB-231 xenograft, and suggesting that CTC profile hides a different message compared to the primary tumor.

### FADS3 is a novel CTC-overexpressed and motility-related determinant of lung metastases in MDA-MB-231 xenograft models

**Fig. 3:**
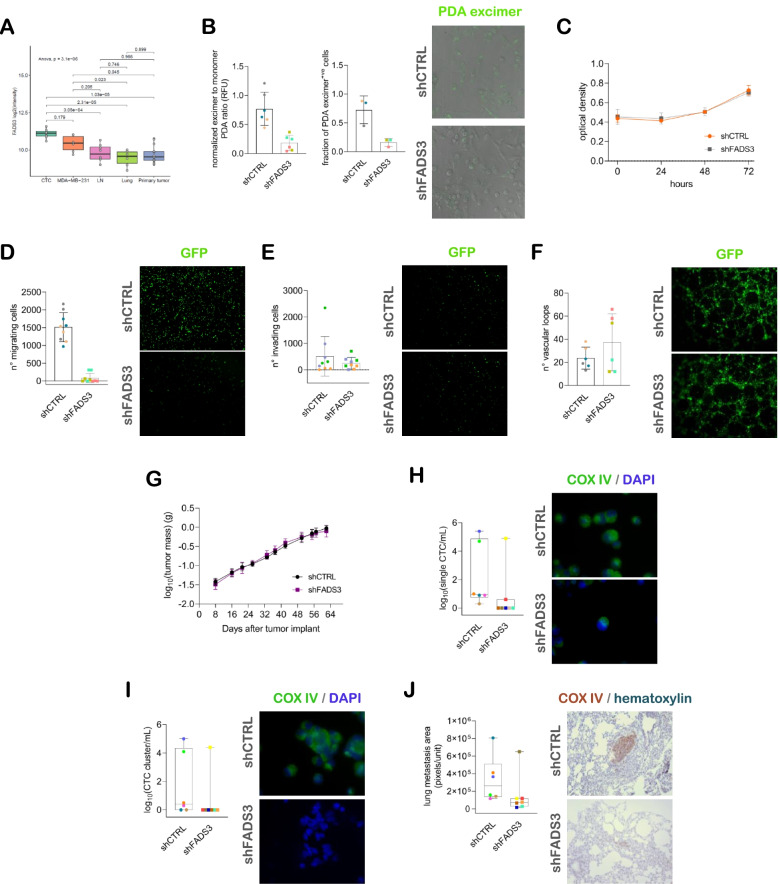
FADS3 regulates MDA-MB-231 cell motility, hematogenous dissemination and lung colonization ability. **A** Box and whiskers plot of *FADS3* expression data (log_2_(signal intensities)) using data from gene expression profile experiments GEP1 and GEP2. **B** (left) Dot plot of the pyrenedecanoic acid (PDA) excimer-to-monomer ratio in a fluorimetric membrane fluidity test, and (right) dot plot of the fraction of PDA excimer positive cells with representative images of fluorescence signals from PDA excimers incorporated at cell membrane level (40x objective). **C** Line chart representation of the mean±standard deviation (SD) optical density measured in a time-course proliferation colorimetric assay (*n*=3 observations in 3 lentiviral infection replicates). **D** (left) Dot plot of the number of migrating cells in a Boyden chamber assay, and (right) representative images of GFP expressing cells at the chamber bottom side (4x objective). **E** (left) Dot plot of the number of Matrigel invading cells (mean of 3 wells) in a Boyden chamber assay (*n*=3 independent experiments with 3 lentiviral infection replicates), and (right) representative images of GFP expressing cells at the chamber bottom side (4x objective). **F** (left) Dot plot of the number of vascular loops, and (right) representative images of GFP expressing cell loops (4x objective). **G** Line chart representation of the mean±SD sum of the tumor masses measured upon cell injection at the inguinal left and axillary right mammary fat pads of NOD SCID mice (*n*=6 shCTRL and *n*=7 shFADS3). **H** (left) Dot plot of single CTC (sCTC) counts, and (right) representative images of COX IV positive CTCs (60x oil immersion objective). **I** (left) Dot plot of CTC cluster (cCTC) count and representative images of (top right) COX IV positive cCTC and (bottom right) a COX IV negative leukocyte cluster (60x oil immersion objective). **J** (left) Dot plot of metastatic foci extent measured in lung sections, and (right) representative images of COX IV positive metastatic cell foci (10x objective)

We screened the list of genes significantly up-regulated (FC≥2 and FDR<0.05) in CTCs compared to primary and secondary solid lesions in both experiments in search of new determinants of metastasis. Within the group of genes related to cell metabolism we noticed a fatty acid desaturase, *FADS3* (Fig. 3A). We hypothesized that FADS3-overexpressing tumor cells have an increased membrane fluidity and motility. We obtained FADS3 stable knock-down cells (Supplementary Fig. S3) and first proved by a pyrene analogue incorporation assay that the activity of FADS3 actually influences membrane fluidity, as shown by the lower distribution of excimer-to-monomer ratio (Fig. 3B, left) and percentage of excimer-positive cells at membrane level compared to control samples (73.0±23.7% versus 16.0±6.7%; Fig. 3B, right). In keeping with its expected role in the membrane fluidity, the proliferation rate remained unvaried between groups over time (Fig. 3C), while FADS3 knock-down impaired the ability to pass through a porous membrane compared to control cells in a migration assay (Fig. 3D), which represents an expected consequence of the higher rigidity of the phospholipidic bilayer. Moreover, consistently with our hypothesis, FADS3 is not involved in other pro-metastatic functions, such as extracellular matrix invasion (Fig. 3E) and vasculogenic mimicry (Fig. 3F), providing further evidence of its specific role in MDA-MB-231 cell motility.

In orthotopic xenograft models, the tumor masses of FADS3 knock-down and control mice were comparable (Fig. 3G), thus corroborating in vitro data. The ability of transplanted cells to disseminate via blood vessels was dramatically reduced upon FADS3 knock-down, in terms of both single CTC (sCTC) and CTC cluster (cCTC) detection rate (3/7 versus 6/6 sCTC+ve, and 1/7 versus 6/7 cCTC+ve, respectively in FADS3 knock-down versus control group) (Fig. 3H, I). Also, the distribution of sCTC number per blood volume was significantly lower in knock-down compared to control mice and showed a trend toward a statistically significant reduction in cCTC number (Fig. 3H, I; *p*-value=0.0332 and *p*-value=0.0495, respectively). Finally, when assessing the total extent of metastatic foci in the lung parenchyma, we found that in the group of FADS3 stable knock-down mice those cells which were able to disseminate displayed a significantly reduced ability to colonize the pulmonary tissue and to give rise to metastatic outgrowth compared to control mice (Fig. 3J; *p*-value=0.0350), confirming the role of FADS3 also during the final phase of the metastatic cascade. Interestingly, nodal involvement was similar in the two groups, and found in all mice, suggesting FADS3 specific role in the hematogenous rather than lymphatic dissemination, and supporting the validation of this gene as a novel CTC-related determinant of lung metastases in the MDA-MB-231 breast cancer model.

### TFF3 is a marker of epithelial-like CTCs involved in MDA-MB-231 migration, invasion and hematogenous dissemination

**Fig. 4:**
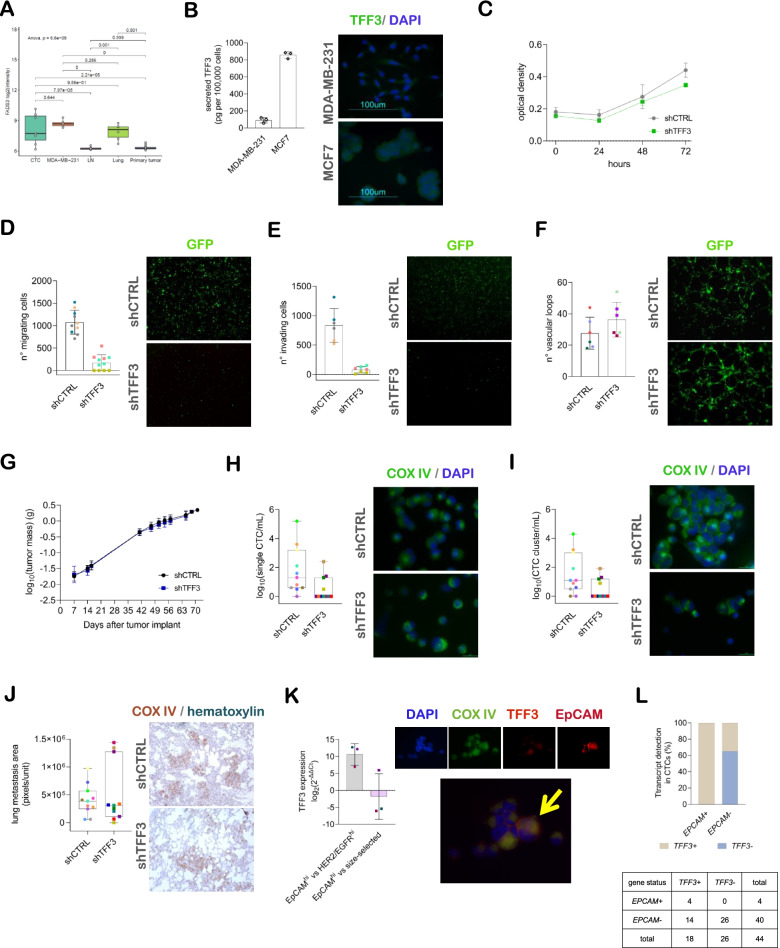
TFF3 is an epithelial circulating tumor cell (CTC) marker involved in MDA-MB-231 migration and dissemination. **A** Box and whiskers plot of *TFF3* expression data (log_2_(signal intensities)) using data from gene expression profile experiments GEP1 and GEP2. **B** (left) Dot plot of the secreted TFF3 concentration in conditioned media (TFF3 quantification in technical triplicates from 3 cultures per cell line), and (right) representative images of intracellular TFF3 positive cells (40x objective). **C** Line chart representation of the mean±standard deviation (SD) optical density measured in a time-course proliferation assay (*n*=3 observations in 3 lentiviral infection replicates). **D** (left) Dot plot of the number of migrating cells in a Boyden chamber assay, and (right) representative images of GFP expressing cells at the chamber bottom side (4x objective). **E** (left) Dot plot of the number of Matrigel invading cells in a Boyden chamber assay, and (right) representative images of GFP expressing cells at the chamber bottom side (4x objective). **F** (left) Dot plot of the number of vascular loops, and (right) representative images of GFP expressing cell loops (4x objective). **G** Line chart representation of the mean±SD sum of the tumor masses measured upon cell injection at the inguinal left and axillary right mammary fat pads of NOD SCID mice (*n*=11 shCTRL and *n*=11 shTFF3). **H** (left) Dot plot of single CTC (sCTC) counts, and (right) representative images of COX IV positive CTCs (60x oil immersion objective). **I** (left) Dot plot of CTC cluster (cCTC) counts, and (right) representative images of COX IV positive cCTCs (60x oil immersion objective). **J** (left) Dot plot of metastatic foci extent measured in lung sections, and (right) representative images of COX IV positive metastatic cells (10x objective). **K** (left) Dot plot of *TFF3* relative expression (FC=(2^-ΔΔCt^); *ACTB* endogenous control) in different CTC subpopulations (EpCAM^hi^: EpCAM-enriched; HER2/EGFR^hi^: HER2/EGFR enriched; size-selected: isolated by filtration), and (right) representative single-fluorescence and merged channel images of a cCTC containing one EpCAM+/TFF3+ tumor cell (*arrow*). **L** (top) Bar chart of *TFF3 *positivity percentage according to *EPCAM* expression in CTC-enriched blood samples of breast cancer patients, and (bottom) 2x2 contingency table of raw values

We searched for other genes involved in the metastatic cascade, with special focus on the hematogenous dissemination phase. To this aim, we considered the list of genes significantly up-regulated in CTC compared to PT samples (log_2_FC≥1.5, FDR<0.0001) and not differentially expressed between CTC and the parental cell line in GEP1 experiment, and we assumed to identify genes expressed in those MDA-MB-231 cell clones which, following the in vivo passage, had acquired a special commitment to metastasis initiation. Within this group of selected genes (Supplementary Table S2), we found the family of trefoil factor secreted peptides *TFF1*, *TFF2* and *TFF3*, which are known to characterize luminal breast cancers. *TFF3* was more expressed in CTCs, lung metastases and the parental cell line compared to PT and LN samples (Fig. 4A). TFF3 peptide was detectable in MDA-MB-231 cells at intracellular and extracellular level, with a mean±SD concentration in the conditioned medium of 90.6±31.0 pg per 100,000 cells (Fig. 4B). We then obtained TFF3 stable knock-down MDA-MB-231 cell models and confirmed the specificity of the shRNA at transcript level within the TFF3 family (Supplementary Fig. S4, panels A, B). Interestingly, whereas TFF3 knock-down did not exert substantial effect on cell proliferation (Fig. 4C), both cell migration and invasion abilities were considerably reduced (Fig. 4D, E), but changes in the vascular mimicry ability were not observed (Fig. 4F). In an attempt to explain the biological role of TFF3 at CTC level, we induced TFF3 transient silencing in MDA-MB-231 cells (Supplementary Fig. S4, panels C, D) and assessed the effect of recombinant human TFF3 (rhTFF3) in functional rescue assays. The proliferation rate did not change and was comparable in both TFF3 silenced cells exposed to rhTFF3 and untreated silenced cells (Supplementary Fig. S4, panel E). Also, the addition of rhTFF3 in the culture medium did not restore the MDA-MB-231 migration ability (Supplementary Fig. S4, panel F). In in vivo functional assays, the tumor growth rate was comparable between the two experimental groups (Fig. 4G), as previously observed in vitro, while both sCTC and cCTC frequencies underwent about 50% decrease in the TFF3 knock-down versus control group (6/11 versus 10/11 sCTC+ve, and 5/11 versus 10/11 cCTC+ve, respectively). Also, the CTC load was significantly lower for both CTC subpopulations in knock-down compared to control mice (Fig. 4H, I; *p*-value=0.021 and *p*-value=0.0418, respectively). Despite the important effect on CTC release, the metastatic burden at pulmonary level was not significantly different between the two experimental groups (Fig. 4J; *p*-value=0.847), indicating that cells disseminated to distant sites reacquired their ability to colonize a foreign microenvironment in the absence of a completely functional TFF3. However, lymph-nodal involvement was observed in all cases in both groups, confirming the specific role of TFF3 in tumor cell spreading via blood vessels rather than via lymphatic system.

Considering the unexpected involvement of TFF3 in the invasive properties of the MDA-MB-231 model, we hypothesized the existence of different CTC subsets and the differential expression of TFF3 among them. Although the majority of MDA-MB-231 cells does not express EpCAM at functional level (Supplementary Fig. S4, panel G), in experiments of antigen-dependent sequential isolation of different types of CTCs we found a subset of EpCAM-enriched cells representing about 1% of the whole population (37±44 out of 3,371±4,598 CTCs, according to the indirect quantification protocol) in MDA-MB-231 xenografts (Supplementary Fig. S4, panel H). Interestingly, *TFF3* showed higher expression in the EpCAM-enriched compared to a HER2/EGFR-enriched CTC subset (Fig. 4K, top) and, in one animal with a total of ~200,000 CTCs we found at least one EpCAM+/TFF3+ cell in 44% of cCTC, which represented the 24% of the whole CTC population, while no double-positive sCTC were observed (Fig. 4K, bottom; antibody species-specificity tests in Supplementary Fig. S4, panel I). Similarly to our experimental models, the relevance of *TFF3* as an epithelial like CTC-associated marker was confirmed in a cohort of 17 M0 and 27 M+ breast cancer patients as *TFF3* was found more frequently expressed in *EPCAM*+ve compared to *EPCAM*-ve CTC-enriched samples collected before starting therapy (Fig. 4L; *p*-value=0.0225). A total of 4 cases showed double positivity and they included two patients with luminal, one with HER2+ve and one with triple-negative tumor.

### Experimentally-derived CTC-specific gene signatures improve the CTC detection rate and predict disease outcome in breast cancers

To assess the clinical relevance of our signature of CTC-upregulated genes derived from the MDA-MB-231 xenograft model, we first tested the specificity of the panel of previously selected 17 genes in a group of 12 female healthy donors. *ADPRHL1*, *ELF3*, *FCF1*, *TFF1*, *TFF2*, *TFF3* and *TPPP* were undetectable in healthy donors (threshold cycle C_q_=40, Supplementary Fig. S5, panel A), while the others were detected at variable frequency and thus they were excluded from our list along with *TFF2* and *TPPP* whose detection frequencies were negligible in the 49 evaluable patients (0 and 1 positive cases, respectively) out of 51 analyzed with AdnaTest. As a result of our algorithm, we identified a signature of 5 CTC-specific genes, i.e., *ADPRHL1*, *ELF3*, *FCF1*, *TFF1* and *TFF3*, and explored its clinical significance according to their expression in CTC-enriched blood samples in our breast cancer case series. We found that the 5-gene panel and AdnaTest detected CTCs in 76% (95% Confidence Interval (CI): 61-87) and 39% (95%CI: 25-55) of evaluable samples, respectively, and that the AdnaTest-positive cases also positive for at least one of the CTC-specific genes were 17 (39%). Moreover, 65.0% M0 and 82.8% M+ patients were defined as CTC-positive by the 5-gene panel compared to 2-fold lower positivity frequencies obtained by AdnaTest (29.4% and 44.8%, respectively for M0 and M+), thus indicating that genes identified in the CTC experimental model are able to increase the positivity rate of the CTC-based test in the clinical context and that epithelial or breast tumor-associated markers might miss some CTC subpopulations. The CTC status assessed by AdnaTest showed an association with the breast cancer molecular subtype (*p*-value=0.0332), whereas by the 5-gene panel we have found no association with the clinico-pathological features considered (Table 1), suggesting that hematogenous dissemination occurs irrespectively of the tumor stage and biological features. We also explored the clinical significance of each individual gene of our CTC-specific signature with respect to the tumor features, and we observed that *TFF1* was associated with the tumor proliferation index and with the histological subtype and was more frequently detected in M+ compared to M0 cases, that also *TFF3* detection frequency was higher in M+ cases, and that *FCF1* was associated to the histological subtype (Supplementary Table S3).

**Table 1:** Association between circulating tumor cell (CTC) status by AdnaTest or by CTC-specific signature and the clinico-pathological features in breast cancers patients

			AdnaTest	AdnaTest+		AdnaTest-			5-gene panel	5-gene panel+	5-gene panel-			
Variable	N	%	N (evaluable)	N	%	N	%	*p*-value	N (evaluable)	N	%	N	%	*p-*value
All patients	51	100.0	46	18	39.1	28	60.9		49	37	75.5	12	24.5	
Age (years)														
<50	11	21.6	10	5	50.0	5	50.0	*.4802*^*a*^	10	9	90.0	1	10.0	*.4143*^*a*^
≥50	40	78.4	36	13	36.1	23	63.9		39	28	71.8	11	28.2	
Clinical stage														
M0	20	39.2	17	5	29.4	12	70.6	*.3011*^*b*^	20	13	65.0	7	35.0	*.1890*^*a*^
M+	31	60.8	29	13	44.8	16	55.2		29	24	82.8	5	17.2	
ER and PgR status														
Positive for either	44	86.3	39	13	33.3	26	66.7	*.0929*^*a*^	42	31	73.8	11	26.2	*.6651*^*a*^
Negative for both	7	13.7	7	5	71.4	2	28.6		7	6	85.7	1	14.3	
HER2/*neu* status														
Positive	9	17.6	8	6	75.0	2	25.0	*.0424*^*a*^	9	6	66.7	3	33.3	*.6693*^*a*^
Negative	42	82.4	38	12	31.6	26	68.4		40	31	77.5	9	22.5	
Subtype														
Luminal	40	78.4	36	11	30.6	25	69.4	*.0646* ^*a*^ *.0332*^*c*^	38	29	76.3	9	23.7	*.4808* ^*a*^ *1*^*c*^
Her2+	8	15.7	7	5	71.4	2	28.6		8	5	62.5	3	37.5	
Triple-negative	3	5.9	3	2	66.7	1	33.3		3	3	100.0	0	0.0	
Histotype														
Ductal	34	66.7	32	13	40.6	19	59.4	*.7366*^*a,d*^	33	26	78.8	7	21.2	*.7098*^*a,d*^
Others	15	29.4	13	4	30.8	9	69.2		14	10	71.4	4	28.6	
Unknown	2	3.9	1	1	100.0	0	0.0		2	1	50.0	1	50.0	
Tumor grade														
G1 or G2	24	47.1	21	7	33.3	14	66.7	*.5671*^*b,d*^	22	17	77.3	5	22.7	*1*^*a,d*^
G3	20	39.2	19	8	42.1	11	57.9		20	16	80.0	4	20.0	
Unknown	7	13.7	6	3	50.0	3	50.0		7	4	57.1	3	42.9	
Ki67														
<10 %	5	9.8	4	2	50.0	2	50.0	*.5840*^*a,d*^	5	4	80.0	1	20.0	*.6431*^*a,d*^
≥10%	30	58.8	26	8	30.8	18	69.2		28	18	64.3	10	35.7	
Unknown	16	31.4	16	8	50.0	8	50.0		16	15	93.75	1	6.25	

^a^ Fisher’s exact test

^b^ Chi-square test

^c^ Luminal versus HER2+/Triple-negative

^d^ “unknown” were excluded from the test

CTC status by any test was not able to predict response to therapy both in M0 and M+ patients. In M0 women, the AdnaTest and the 5-gene panel detected 23% versus 63% of cases who did not reach pathologic complete response (pCR; Table 2) following neoadjuvant therapy. In M+ women, the 5-gene panel detected 100% of patients with stable disease (SD) and progressive disease (PD), whereas the AdnaTest only 38%, but both tests gave positive result for 69% and 53% of patients with complete response (CR) and partial response (PR) according to RECIST (Table 2).

**Table 2:** Association between CTC status by AdnaTest or by CTC-specific signature and response to systemic therapy in breast cancer patients

		AdnaTest	AdnaTest+		AdnaTest-			5-gene panel	5-gene panel+		5-gene panel-		
Variable	N	N (evaluable)	N	%	N	%	*p*-value	N (evaluable)	N	%	N	%	*p*-value
Pathologic response (M0)	20	17	5	29.4	12	70.6		20	13	65.0	7	35.0	
Responder (pCR)	2	2	2	100	0	0	*.0952*^*a,c*^	2	2	100	0	0	*.5294*^*a,c*^
Non responder	16	13	3	23.1	10	76.9		16	10	62.5	6	37.5	
Unknown	2	2	0	0	2	100		2	1	50	1	50	
RECIST response (M+)	31	29	13	44.8	16	55.2		29	24	82.7	5	17.2	
CR	1	1	0	0.0	1	100.0	*.6668*^*a,c,d*^ *1.0*^*a,c,e*^	1	0	0.0	1	100.0	*.1304*^*a,c,d*^ *.0705*^*a,c,e*^
PR	16	14	8	57.1	6	42.9		15	11	73.3	4	26.7	
SD	1	1	0	0.0	1	100.0		1	1	100.0	0	0.0	
PD	7	7	3	42.9	4	57.1		7	7	100.0	0	0.0	
Unknown	6	6	2	33.3	4	66.7		5	5	100.0	0	0.0	

*M0* patients with early-stage breast cancer, *M+* patients with metastatic breast cancer, *pCR* pathologic complete response, *CR* complete response, *PR* partial response, *SD* stable disease, *PD* progressive disease, *RECIST* Response Evaluation Criteria in Solid Tumors

^a^ Fisher’s exact test;

^b^ Chi-square test;

^c^ “unknown” were excluded from the test

^d^ CR/PR vs SD/PD

^e^ CR vs PR/SD vs PD

**Fig. 5:**
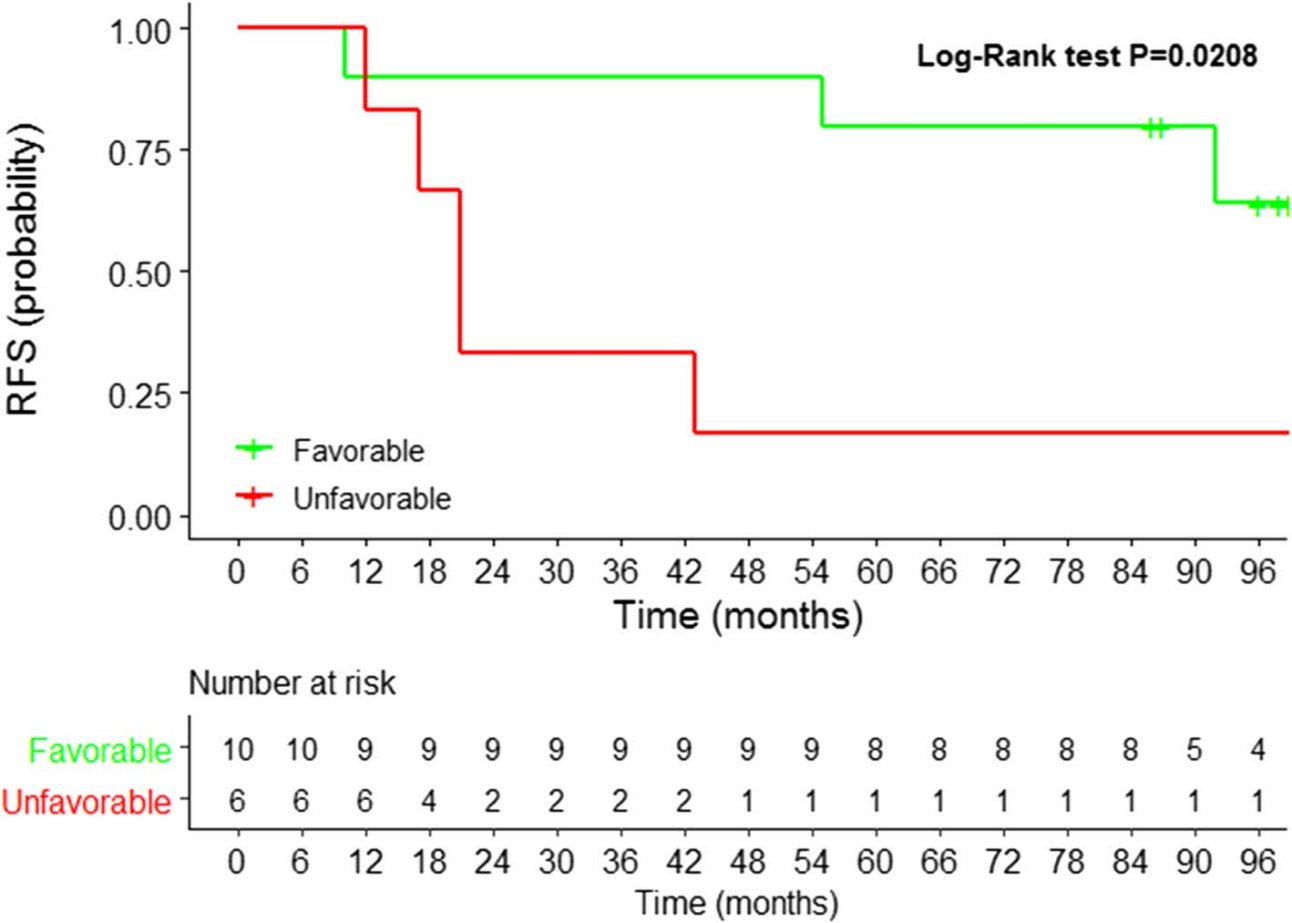
Circulating tumor cell (CTC) kinetics inform prognosis in breast cancer patients subjected to neoadjuvant therapy. Kaplan-Meier plot of eight-year Relapse-free survival (RFS) probability in M0 breast cancer patients according to CTC kinetics from the start of systemic treatment to an early time point during therapy administration. Unfavorable: positive-positive CTC trend from T0 (before therapy administration) to T1 (during therapy); Favorable: negative-negative, positive-negative, negative-positive CTC trend.

As concerns prognostic endpoints, the clinical setting (M0 vs M+) was significantly associated with event-free survival (i.e., distant relapse or progression respectively for M0 and M+) with an Hazard Ratio (HR) of 0.23 (95%CI: 0.10-0.52) (Supplementary Fig. S5, panel B). Neither AdnaTest nor the 5-gene panel were associated with prognosis within each setting when CTC status was assessed at baseline (T0), whereas the prognosis of M0 cases with unfavorable CTC trend, i.e., with positive CTC status both at T0 and T1 (during therapy) according to the 5-gene panel - overall 9 out 17 M0 positive out of CTC evaluable cases at T1, and 6 positive at both time points - was significantly different compared to the counterpart with a more favorable CTC trend (HR 4.67, 95%CI: 1.06-20.61; Supplementary Table S4, Fig. 5), indicating that the CTC kinetics rather than the CTC status at baseline, as assessed by the expression analysis of CTC-specific genes, can predict distant relapse in the neoadjuvant setting.

## Discussion

In this study we show that cancer cell hematogenous dissemination in the MDA-MB-231 xenograft model is driven by a massive transcriptional reprogramming, which implies the downregulation of numerous genes subtending the remodeling of chromatin and the regulation of transcription, and which determines the up-regulation of genes involved in several biological processes mainly related to cell plasticity and adaptation. We report that primary tumor nodules and lung and lymph-node metastases share a common transcriptional context, whereas cells endowed with the ability to systemically spread possess a distinct gene expression profile. Consistently, in the CTC population of breast cancer patients we have observed a trend toward higher expression level of a panel of genes up-regulated in experimentally-derived CTCs compared to tumor cells biopsied from matched primary lesions. We also demonstrate that the fatty acid desaturase *FADS3*, one of the genes overexpressed in our CTC model, represents a novel CTC-related metastasis-promoting gene, responsible for cell membrane fluidity - as it encodes an enzyme that catalyzes double bond introduction into the fatty acid acyl chains, a chemical modification that determines the level of phospholipids packing - motility, dissemination via blood vessels, both as single or clustered CTCs, and metastatic colonization of the lungs but not the lymph-nodes. *FADS3* was also more expressed in CTC-enriched blood samples compared to tumor cell-enriched fine-needle biopsies in a group of breast cancer patients. However, we did not observe differential expression between patients and healthy donors when *FADS3* was measured in blood samples. Also, the luminal breast cancer-related gene *TFF3* was found to be up-regulated in CTCs and lung metastasis compared to primary tumor and lymph-node metastases, and associated to an epithelial-like CTC phenotype in the experimental model and in breast cancer patients. Finally, we have identified a CTC-specific gene-panel which increases the sensitivity of CTC-based tests both in early stage and metastatic breast cancers with no association to the clinico-pathological features of the case series. Notwithstanding the model from which we originated the signature is negative for hormone receptors and HER2 amplification, our CTC-specific gene panel was able to predict the risk of relapse also in luminal breast cancers. Actually, the CTC kinetics as assessed by our panel of genes identified patients at higher risk of distant relapse in the neoadjuvant setting.

To the best of our knowledge, studies extensively describing the gene expression profile of CTCs in the MDA-MB-231 and other breast cancer experimental models have not been published, yet. Here, we extend prior research on CTCs in experimental models with breast cancer cell lines showing that cancer cell hematogenous spread is a step of the metastatic cascade, governed by the activation of a transcriptional program peculiar to CTCs in the MDA-MB-231 model, and which involves several biological processes including also adaptation to external stimuli and plasticity. As opposite to CTCs, no sub-clusters of samples belonging to the same tumor tissue emerged within the principal cluster of solid tumor lesions when analyzing gene expression data, thus indicating that primary tumor nodules and metastases shared similar expression patterns, which instead were distinct from those peculiar to disseminated cells, and also suggesting a tendency of tumor cells to restore the molecular profile of the primary tumor after completing the hematogenous dissemination phase and colonizing lymph-nodal and pulmonary tissues.

By performing functional studies, we reported data on the involvement of two CTC upregulated genes as metastasis promoters. Literature data on the role of FADS3 in breast cancer are still scanty [25], although evidence for an altered fatty acid transport, synthesis and desaturation was recently reported as responsible for breast cancer response to therapy, recurrence and plasticity [26–28]. In this study we focused on the effect of fatty acid desaturation on membrane fluidity and demonstrated that CTCs exploit the enzymatic activity of FADS3 to increase their motility, with no influence on cell proliferation and tumor growth rate in MDA-MB-231 models. Although *FADS3* was not listed among genes classified as CTC-specific according to our observations in healthy donors, several endpoints of fatty acid metabolism have been long considered as possible therapeutic targets. In fact, there is strong rationale for their involvement in tumor progression [29], and trials with inhibitors of fatty acid metabolism are ongoing [30, 31].

We have also explored the function of *TFF3*, one of the genes up-regulated in CTCs compared to the primary tumor, which is known to characterize the luminal breast cancer subtype. In the past, *TFF3* - and *TFF1*, but not *TFF2* - mRNA was detected in breast tumors and estrogen-responsive breast cancer cell lines [32–34] and *TFF3* was ranked among genes down-regulated in MDA-MB-231 compared to MCF7 cells [35]. Differently from published data, our research demonstrates that TFF3 is detectable at mRNA level and as a secreted peptide in MDA-MB-231 cells in vitro, although higher expression was observed when comparing EpCAM-enriched to HER2/EGFR-enriched CTCs, consistently with its frequent detection in the luminal and more epithelial-like breast cancers. Moreover, in line with this evidence, in previous studies we found that TFF3 is significantly overexpressed in MCF7-derived mammospheres compared to the parental cell line [36, 37]. *TFF3* was undetectable in healthy donor blood samples and more frequently detected in the CTC fraction of breast cancer patients with advanced compared to early-stage disease, similarly to other reports demonstrating an association with breast cancers progressed to bone metastases [38] and leptomeninges [39], and listing *TFF3* among those genes belonging to a specific genomic region that frequently undergoes copy number gain at CTC level in the metastatic setting [40]. A multi-marker panel including both *TFF1* and *TFF3* revealed that *TFF3* was 10 to 15-fold more expressed in the peripheral blood mononuclear cell fraction isolated from patients with metastatic breast cancer compared to healthy controls [41]. Interestingly, studies on the dynamic changes of CTCs in the epithelial and mesenchymal composition showed that tumor cells with epithelial features, which were highly representative of the CTC population in ER-positive/PgR-positive breast cancer cases and persistent in patients with initial response to therapy, overexpressed *TFF1* and *TFF3* compared to tumor cells with mesenchymal or intermediate features [42]. Although aberrant expression of TFF3 has been reported for a variety of tumors [43], data on its specific role depict it as a molecule acting in a tumor type- or context-dependent manner; more importantly, the receptor for TFF3 has not been discovered or validated, yet [44]. Since in our experiments TFF3 knock-down did not influence MDA-MB-231 vascular mimicry ability, contrarily to other reports describing TFF3 as a promoter of tumor angiogenesis in breast cancer cells [45], the exposure to recombinant TFF3 did not restore the migratory ability in TFF3-silenced MDA-MB-231 cells, possibly due to a major role for intracellular rather than extracellular TFF3. Considering current knowledge of TFF3 biological activity, further studies are needed to clarify its mechanism of action in CTC dissemination.

The identification of breast cancer biomarkers by high-throughput molecular analyses has rapidly increased in the latest years. In the perspective of a personalized approach to patient monitoring and treatment, assessing the clinical relevance of CTC-related biomarkers, which means looking at CTCs as a boundless source of information and not only as a discrete and countable marker, might improve our ability to predict outcome. In fact, a CTC-signature, recently derived from gene expression comparative analysis between breast tumor or normal tissues and blood samples, was associated with higher probability of residual disease at surgery in a cohort of localized breast cancers when assessed at CTC level [46]. Analyzing M0 and M+ breast cancer clinical samples, we found that the detection of at least one among five CTC-specific genes before starting primary systemic treatment increased the CTC-detection rate compared to standard CTC-related markers. Our CTC test did not predict response to therapy as assessed at surgical resection or at radiological evaluation. However, with the 5-gene panel we detected a higher number of stage IV patients who did not respond to systemic therapy compared to the CTC-test based on the expression of conventional epithelial and tumor-associated markers. These results are also consistent with the high frequency of CTCs we have observed during the course of a neoadjuvant therapy or a first line therapy for metastatic disease, and with the observation that CTC fluctuations, as assessed by gene expression analysis, can mirror the clinical situation with higher accuracy compared to other markers and/or CTC status assessed at baseline only, as also corroborated by our previous studies in other cancer types [47, 48].

Studies with experimental models that mirror breast cancer heterogeneity, such as patient-derived primary tumor xenografts or CTC cultures, as also validation studies on larger case series and using technologies that ensure CTC capture at single cell resolution are still needed to solve the tight regulation of hematogenous spread during cancer progression. Our work prove the importance of analyzing CTCs taking into account the cellular context at primary and secondary tumor sites and demonstrate that CTC-specific genes with proven biological role in the metastatic cascade are more relevant to the clinical setting than standard epithelial and breast tumor-associated markers and improve our ability to develop reliable tests for disease monitoring.

## Conclusions

In conclusion, hematogenous dissemination is regulated by numerous genes. Our new CTC signature derived from a breast cancer xenograft model improved CTC detection and outcome prognostication in early-stage patients compared to conventional CTC markers, and shed light on the metastatic process by highlighting the role of two genes: *FADS3* and *TFF3*. We propose that the application of a comprehensive approach based on the comparison between CTC and solid lesion gene profiles, integrated by functional validation and mechanistic preclinical studies, could improve knowledge of blood-borne cancer dissemination and allow the identification of new clinically promising signatures.

## Supplementary Information

**Additional file 1: Supplementary information.** File includes extended Methods section, 5 Figures and 6 Tables.

**Additional file 2: Supplementary File S1.** File contains the list of genes differentially expressed in circulating tumor cells versus solid lesions in MDA-MB-231 xenograft models.

**Additional file 3: Supplementary File S2.** File reports results of gene ontology enrichment analysis using genes up-regulated in circulating tumor cells versus solid lesions in MDA-MB-231 xenograft models.

**Additional file 4: Supplementary File S3.** File reports results of gene ontology enrichment analysis using genes down-regulated in circulating tumor cells versus solid lesions in MDA-MB-231 xenograft models.

## Data Availability

The gene expression profile data generated in this study are publicly available at NCBI Gene Expression Omnibus (GEO) repository with accession number GSE188899, within the article and its supplementary data files. All data generated or analyzed during this study are included in this published article and its supplementary information files.

## Abbreviations

ADPRHL1: ADP-ribosylhydrolase like 1
CI: Confidence interval
COX IV: Cytochrome c oxidase complex IV
CR: Complete response
CRIP1: Cysteine rich protein 1
CTC: Circulating tumor cell
DTC: Disseminated tumor cell
EGF3: Epidermal growth factor receptor
ELF3: E74 like ETS transcription factor 3
EpCAM: Epithelial cell adhesion molecule
FADS3: Fatty acid desaturase 3
FC: Fold change
FCF1: FCF1 rRNA-processing protein
FDR: False discovery rate
FIS1: Fission mitochondrial 1
GAS2L1: Growth arrest specific 2 like 1
GEP: Gene expression profile
GFP: Green fluorescent protein
GO: Gene ontology
HDAC10: Histone deacetylase 10
HER2: Human epidermal growth factor receptor 2
HR: Hazard ratio
IQR: Interquartile range
KLC2: Kinesin light chain 2
LN: Lymph-node metastases
LUNG: Lung metastases
M+: Metastatic
M0: Non-metastatic
MET: MET proto-oncogene, receptor tyrosine kinase
MUC-1: Mucin 1
NOD SCID: Non-obese diabetic/severe combined immunodeficiency
NR4A1: Nuclear receptor subfamily 4 group A member 1
OC: Overlap coefficient
pCR: Pathologic complete response
PD: Progressive disease
PDA: Pyrenedecanoic acid
PR: Partial response
PT: Primary tumor
PTPRC: Protein tyrosine phosphatase receptor type C
RECIST: Response evaluation criteria in solid tumors
RFS: Relapse-free survival
SD: Stable disease
SD: Standard deviation
shRNA: Short hairpin RNA
STRN4: Striatin 4
TAF6: TATA-box binding protein associated factor 6
TFF1: Trefoil factor 1
TFF2: Trefoil factor 2
TFF3: Trefoil factor 3
TPPP: Tubulin polymerization promoting protein
