# Supplementary material for: Gene signatures of circulating breast cancer cell models are a source of novel molecular determinants of metastasis and improve circulating tumor cell detection in patients
Source: J Exp Clin Cancer Res. 2022 Feb 25;41:78. doi: 10.1186/s13046-022-02259-8 (PMC8876758; doi:10.1186/s13046-022-02259-8)
Supplement: Supplementary file 1 — Additional file 1: Supplementary information. File includes extended Methods section, 5 Figures and 6 Tables. [file 13046_2022_2259_MOESM1_ESM.pdf]

SUPPLEMENTARY INFORMATION

Supplementary Methods

Cell lines and spike-in experiments

MDA-MB-231 (HTB-26^{TM}) and MCF7 (HTB-22^{TM}) human breast cancer cell lines were cultured in Dulbecco’s Modified Eagles’s Medium (DMEM)/F-12 (Lonza, Switzerland) supplemented with South America FBS (Lonza) at a 5% and 10% concentration, respectively. HEK293T (CRL-3216^{TM}) human embryonic kidney, 4T1 (CRL-2539^{TM}) murine breast cancer, and CT26 (CRL-2638^{TM}) murine colon cancer cell lines were cultured in DMEM supplemented with 10% FBS and 1 mM L-Glutamine (Lonza). HUVEC (CRL-1730^{TM}) cells were cultured in Endothelial Cell Growth Medium supplemented with EGM™-2 BulletKit™ (Lonza). All cell lines were purchased from the American Type Culture Collection (ATCC; Manassas, VA, USA) and propagated under controlled 37°C, 95% humidified and 5% pCO_2 atmosphere for not more than 7 passages. Cells were used for experiments after the second passage from thawing. MDA-MB-231 and MCF7 cell lines were authenticated via short tandem repeat profile analysis with the StemElite™ ID System kit (Promega) according to the ATCC guidelines. Cells were periodically tested for mycoplasma contamination by the MycoAlert® detection kit (Promega, Madison, WI, USA) on the GloMax® 20/20 luminometer and re-tested before every xenograft experiment. Mycoplasma contamination was never observed.

Spike-in experiments were performed by injecting single MDA-MB-231 cells pipetted under an inverted microscope from highly diluted cell suspensions in culture dish directly into 5-mL blood samples donated from healthy volunteers.

Mouse models and xenograft experiments

Female NOD.CB17-*Prkdc^{scid}*/NCrCrl (NOD SCID; Strain Code: 394) mice were purchased from Charles River (Wilmington, MA, USA), and bred by qualified personnel at Fondazione IRCCS Istituto Nazionale dei Tumori (in Milan, INT) Animal House Facility in individually ventilated cages, 3 to 5 animals per cage. Animals were anesthetized by intraperitoneal injection of ketamine (100 mg/kg) and xylazine (5 mg/kg) cocktail before orthotopic injection of cancer cells and before animal sacrifice. All procedures were performed under sterile conditions. All the in vivo experiments were conducted blindly with respect to the type of experimental group, i.e. injection with control or gene knock-down cells. Tumor implant was performed on healthy and normal-weight 7- to 16-week-old anesthetized female NOD SCID mice using a 30G needle syringe. Eighty- to 90-µL Dulbecco's PBS (DPBS, Lonza, Switzerland) cell suspensions of $5x10^6$ cells each mixed with 50% ECM Gel from Engelbreth-Holm-Swarm murine sarcoma Matrigel (Sigma-Aldrich, Saint Louis, Missouri, USA) matrix (final concentration 4 mg/mL) were injected into the mammary fat pad of the right axillary and the left inguinal mammary glands. Tumor growth was monitored every week using a caliper and the tumor mass (g) was estimated by the $(D \times d^2)/2$ formula, where D and d represent the longest and the shortest diameter of the nodule,

respectively. The tumor load did not exceed 10% of body weight. Blood samples (up to 1 mL) were drawn from anesthetized mice by cardiac puncture, using a 26G needle EDTA-conditioned syringe (Sigma-Aldrich, final concentration in blood 1.8 mg/mL), stored at 4°C and processed within 30 minutes for circulating tumor cell (CTC) isolation [18]. Mice were immediately sacrificed and axillary and inguinal primary tumor nodules, lungs, and axillary and/or inguinal lymph-nodes (homolateral and/or contralateral to the primary tumor) were collected and fixed in 10% neutral buffered formalin solution (Bio-Optica, Milan, Italy) for 18-24 hours or embedded in Compound embedding medium for cryostat (O.C.T., Bio-Optica) and snap frozen in liquid nitrogen. Bone marrow cells were flushed out from femurs with DPBS and processed for the isolation of tumor cells (bone marrow disseminated tumor cells, DTCs) within 30 minutes. Sacrifice procedure was cervical dislocation, performed at fixed time points or upon first signs of moderate suffering (e.g., decrease in activity, hunched appearance, ruffled hair coat, respiratory distress). Animal experiments were performed according to the Italian law D.L. 116/92, and following additions, which enforced the 2010/63/EU Directive. The study protocols were approved by the Ethics Committee for Animal Experimentation at INT (INT_08/2012, and INT_01/2017, which was also approved by the Italian Ministry of Health with approval number 452/2017-PR, following the receipt of D.L. 6/2014). Studies were designed according to the 3Rs criteria, and all efforts were deployed to minimize animal suffering [19], following the most recently published version of recommended ARRIVE guidelines (https://www.nc3rs.org.uk/arrive-guidelines). The sample size for gene expression profile experiments was calculated taking into account the CTC numbers obtained in previous exploratory tests in order to ensure a minimum number of three animals with at least 5,000 CTCs per experiment. The sample size for metastasis assays was estimated taking into account the variability in CTC detection and extent of pulmonary metastases in preliminary experiments. For each experiment, animals were taken from the same batch and were assigned to the various experimental groups by simple random sampling (random numbers generator www.random.org). The numbers of animals and of biological samples per experimental group which were used or were available for CTC and metastasis assays were reported in **Supplementary Table S5**. Briefly, we used the minimum number of animals sufficient to obtain reliable data based on previous experiments with MDA-MB-231 cells and considering the CTC load and the presence of pulmonary metastases as biological endpoints. For functional assays to investigate the role of *TFF3*, we expected higher variability due to the poor association of this gene with triple-negative breast cancers, and increased the number of animals accordingly.

Patients and donors

Patients with histologically confirmed diagnosis of breast cancer and no evidence of metastatic disease (M0, N=20) candidate to neoadjuvant therapy (anthracycline/taxane (AT) plus trastuzumab if HER2-positive), and breast cancer patients with stage IV disease (M+, N=31) who were starting a first line of systemic treatment (AT and cyclophosphamide/methotrexate/fluoruracil (CMF) or carboplatin and paclitaxel for hormone receptor negative, AT/CMF and trastuzumab if HER2-positive, or AT/CMF and hormone therapy or vinorelbine and capecitabine if hormone receptor positive) were prospectively enrolled from

December 2010 to June 2013 at the Department of Medical Oncology at INT. Blood samples were collected from each patient before starting treatment, and at subsequent time points (before initiation of taxane-based neoadjuvant therapy in M0 cases, and during the course of treatment in M+ cases) when possible according to patient health status. Pathologic complete response (pCR) was defined as the absence of cancer cells in the surgical specimens of breast and lymph nodes following completion of neoadjuvant therapy. Response to therapy in M+ cases was assessed by radiological evaluation according to Response Evaluation Criteria in Solid Tumors (RECIST 1.1; complete response, CR: tumor disappearance; partial response, PR: 30% or greater decrease in longest diameter; PD: 20% or greater increase in longest diameter; stable disease, SD: tumor with no sufficient shrinkage or increase to be classified as PR or PD, respectively). Fine-needle aspirates were collected from 4 tumor nodules (2 M0 cases and 2 patients with subsequent diagnosis of metastatic disease) during routine diagnostic or surgical procedures. Female healthy subjects were recruited during spontaneous blood donation at ADSINT (Associazione Donatori di Sangue – INT). Study protocols were approved by the Institutional Review Board and the Ethics Committee at INT (INT-13/10/2010 – 0003703 and following amendment). All patients and healthy subjects signed an informed consent for blood sample donation dedicated to this research.

Circulating tumor cell isolation, detection and enumeration

In xenograft models, CTCs and DTCs were isolated using the ScreenCell® Cyto kit (ScreenCell, Sarcelles, France). Briefly, blood was diluted in DPBS-EDTA (1.8 mg/mL) to reach 3 mL and mixed with 4 mL of ScreenCell® FC2 buffer for red blood cell osmotic lysis and cell fixation. CTC clusters were defined as aggregates (juxtaposed cell membranes) of at least 2 CTCs [18]**.** Cells immobilized on ScreenCell® isolation supports were stained for CTC detection or immunofluorescence analysis. Blood samples with number of CTCs exceeding the filtration capacity of the isolation support (i.e., when the density of cells with diameter >>8 μm was higher than 1,000 cells per filtration volume and the blood flux through the filter stopped due to pore saturation) were diluted in DPBS 1X and processed with additional filters for direct CTC count, or centrifuged to collect cell pellets and estimate CTC concentration in a Bürker chamber under an inverted microscope (CTCs were recognized on the basis of the whole cell size, about 5 to 10-fold higher compared to murine leukocytes, in N=3 xenograft models). The absolute number of CTCs was normalized on the blood volume.

For gene expression profile experiments, CTCs were isolated using two sequential approaches: 1) blood samples were incubated with 50 μL of magnetic beads coated with antibodies against EpCAM, MUC1, HER2 and EGFR from AdnaTest® EMT-2/StemCell Select kit (AdnaGen AG, Langenhagen, Germany) and bead-bound cells were captured using the AdnaMag magnet, then 2) residual blood was filtered using the ScreenCell® MolecularBiology kit which enables cell collection in capsule-filters. For sequential capture of distinct CTC subpopulations from the same animal model, 1) blood samples were sequentially incubated with magnetic beads coated with antibodies against a) EpCAM (Berp4) and b) HER2 and EGFR (kindly provided by Dr. Siegfried Hauch from AdnaGen), and then 2) residual blood was filtered with the ScreenCell® MolecularBiology kit. Cells were immediately lysed with the AdnaTest Lysis Buffer, lysates were stored at -20°C until mRNA isolation (up to 14 days), and CTC number was estimated by quantitative real time PCR as described below.

In patients and healthy donors, CTCs were isolated from 5 mL of peripheral venous blood, collected in K_2EDTA BD Vacutainer tubes after discarding the first milliliter of blood to minimize the contamination from skin cells. Samples were stored at 4°C and processed within 1 hour for CTC analysis. CTC enrichment and tumor cell isolation from fine-needle biopsies in breast cancer patients were performed using magnetic beads coated with antibodies against EpCAM and MUC1 available in the AdnaTest® EMT-1/StemCell Select kit (AdnaGen). Blood samples of healthy donors (N=12) were processed using magnetic beads coated with antibodies against EpCAM, MUC1, ErbB2 and EGFR, according to the AdnaTest® EMT-2/StemCell Select kit (AdnaGen) protocol, with the intent to increase the specificity of CTC positivity cut-off values in patients as the improved cocktail is able to ameliorate the capture of CTC subpopulations compared to AdnaTest® EMT-1/StemCell Select kit [20]. CTC positivity by AdnaTest markers was assessed using a multiplex PCR protocol for the expression of *EPCAM*, *MUC1*, *ERBB2*, *PIK3CA*, *AKT2*, *TWIST1* and *ALDH1A*, according to the AdnaTest® EMT-1/StemCell Detect kit, using positivity cut-off values and quality control criteria previously described [20].

For spike-in experiments, the capture of MDA-MB-231 cells was assessed by semiquantitative PCR for *MET* using the AdnaTest workflow and specific primers provided by AdnaGen.

PCR products were run on the Agilent 2100 Bioanalyzer System using the Agilent DNA1000 kit (Agilent Technologies, Santa Clara, CA, USA).

Tumor cell isolation from fine-needle biopsies

Fine-needle biopsies were collected in sterile tubes containing 5 mL of DMEM/F-12 FBS supplemented culture medium, and processed within 1 hour with the same protocol used for CTC detection by AdnaTest® EMT-1/Stem CellSelect kit, in order to minimize selection biases between different sample sources and make matched CTC and primary tumor sample profiles comparable.

Transient gene silencing with siRNA molecules and functional rescue experiments

MDA-MB-231 cells were seeded at a 70% cell density and transfected after 24 hours with the *TFF3* Trilencer 27mer Human siRNA "GAUGUCUUAACGAAUAAAGGUCCCA" and the Scrambled Negative Control siRNA Duplex (Origene, Rockville, MD, USA) at a concentration of 25 nmol/L, using the TransIT-X2® Dynamic Delivery System (Mirus Bio LLC, Madison, WI, USA), according to the manufacturer's standard protocol. Delivery efficiency after 48 hours was 75% as assessed by transfecting cells with the BLOCK-iT™ Fluorescent Oligo labeled with Cy3 (Thermo Fisher Scientific, Waltham, MA, USA). For proliferation and migration rescue experiments, cells were incubated with recombinant human TFF3 peptide (rhTFF3, Origene) at a concentration of 1 ng/mL and seeded for functional assays after 24 hours from transfection. Red-fluorescent HUVEC cells stained with the CellTracker^{TM} Deep Red fluorescent dye were exposed to 2.5 ng/mL rhTFF3 and tracked for migration in a Transwell® assay as positive control of rhTFF3 stimulation based on previous reports [45].

Lentivirus-mediated long-term stable gene knock-down

MDA-MB-231 cells were seeded in triplicate in 6-well plates at a 70% cell density and infected after 24 hours with lentiviral particles delivering three 29mer human *TFF3* or *FADS3* shRNA provided by the manufacturer (Origene, catalog numbers TL318798V and TL304708V,

respectively), one shRNA per cell plate. Gene knock-down efficiency was tested at mRNA and protein level. Functional assays were performed using lentiviral particles delivering the shRNA with sequence "GTGATTGCTGCCAGGCACTGTTCATCTCA" for TFF3 or "GTGACATCTGGCTGGACGCCTACCTCCAT" for FADS3 stable knock-down, and non-effective 29-mer Scrambled shRNA Cassette as control, in a pGFP C-shLenti vector, at a multiplicity of infection of 5. For TFF3 knock-down experiments, cells were infected with commercially available lentiviral particles (Origene). For FADS3 knock-down experiments, *FADS3* shRNA lentiviral particles (Origene) were previously packaged in HEK293T cells (ATCC). Infection efficiency was about 70% as observed under a Nikon Eclipse TE2000-S fluorescence microscope (Nikon Europe B.V., Amsterdam, Netherlands). After 48 hours the culture medium was changed and cells were put under constant selection with 0.5 µg/mL Puromycin (Sigma-Aldrich, Saint Louis, Missouri, USA) versus not infected controls until death of all control cells (10 days).

Proliferation assay

MDA-MB-231 cell proliferation rate was assessed using the CellTiter 96® AQueous One Solution Cell Proliferation Assay (MTS, Promega). For transient silencing experiments, cells were previously seeded in 24-well plates at a 70% confluence and transfected for 48 hours. Cells were then seeded in 96-well plates at a 20% confluence and after 12, 24, 48 and 72 hours they were incubated with the MTS reagent in a culture chamber. After 2 hours the reaction was stopped with SDS 10%, the optical density (O.D.) was measured at a 490 nm absorbance wavelength by the iMark™ Microplate Absorbance Reader spectrophotometer (Biorad, Hecules, CA, USA) and O.D. values were obtained by the Microplate Manager 6 software and corrected for blank. MTS assays with TFF3 or FADS3 stable knock-down models were directly run in 96-well plates after 24 hours from seeding.

Migration and invasion assays

MDA-MB-231 migration and invasion ability was assessed using a Boyden chamber assay. Cells were seeded in serum-free medium on 6.5-mm diameter Transwell® membrane cell culture inserts (6.0 x 10^4 cells per insert) with 8.0 µm pore diameter (Corning®, New York, USA). For invasion assays, Transwell® membranes were previously coated with BD Matrigel™ Basement Membrane Matrix Growth Factor Reduced (BD Biosciences, San Jose, CA, USA) at a 200 µg/mL concentration. Cell chemotaxis was stimulated after 24 hours from seeding by using complete growth medium as an attractant in the bottom well. Cells on the top of the membrane were removed after 36 hours using a cotton-tipped applicator, washed in DPBS (Lonza), fixed in ethanol absolute for 20 minutes at -20°C and stained with 0.4% sulforhodamine B (Sigma-Aldrich) for 30 minutes at room temperature, or directly observed under a 4x objective with a Nikon fluorescence inverted microscope if expressing GFP. The whole insert was digitalized by NIS-Elements imaging software (Nikon Europe B.V.), and migrating or invading cells were counted using the ImageJ software version 1.51g (https://imagej.nih.gov/ij/).

Membrane fluidity assay

Membrane fluidity was assessed using the MarkerGene™ Membrane Fluidity kit (Marker Gene Technologies, Inc., Eugene, OR, USA). Briefly, cell suspensions or cells cultured in µ-Slide

8-well chambers (ibidi GmbH, Gräfelfing, Germany) were incubated with 10 µM pyrenedecanoic acid (PDA) for 20 minutes at room temperature and then at 37°C in a culture chamber. Fluorescence emitted by PDA excimers and monomers was read after 30 minutes with the FLUOstar® OPTIMA plate reader (BMG Labtech, GmbH, Offenburg, Germany) by exciting at 400 nm and measuring monomer and excimer emission with 492 nm and 570 nm filters, respectively. Direct visualization of fluorescence signals in cultured cells was performed using the Leica TCS SP8 X confocal laser scanning microscope (Leica Microsystems GmbH, Mannheim, Germany) by exciting with a continuous wave 405 nm diode laser and detecting monomer fluorescence from 410 to 422 nm and excimer fluorescence from 450 to 550 nm.

Vascular mimicry assay

Cell suspensions of 4.0 x 10^5 cells/mL were prepared in serum-free medium and 2.0 x 10^4 cells were seeded onto 96-well plates coated with pure Corning® Matrigel® Basement Membrane Matrix (Corning), which was kindly provided by the Molecular Targeting Unit at INT. Loop formation was monitored under a 4x objective with a Nikon TE-2000-S fluorescence inverted microscope. Wells were digitalized and vascular loops were manually counted after 6-8 hours.

RNA extraction and cDNA synthesis

For CTC indirect quantification and gene expression profiling experiments, mRNA was isolated from cell lysates using the AdnaTest® EMT-2/Stem CellDetect kit (oligo(dT)25-coated Dynabeads®) according to the manufacturer protocol, and diluted in 10 µL of nuclease-free not-DEPC treated water (Ambion, Thermo Fisher Scientific, Waltham, MA, USA). Half RNA volume was used for tumor cell indirect quantification, and the remaining volume was stored at -80°C for microarray gene expression profile analyses. Total RNA was extracted from cell pellets or from 10-µm-thick frozen sections of O.C.T.-embedded tumors, lungs and lymph-nodes through the *Agencourt*® RNAdvance™ *Tissue* Kit (Beckman Coulter, Breas, CA, USA), according to the manufacturer's instructions, and quantified at NanoDrop™ UV-Vis Spectrophotometer (Thermo Scientific™). RNA quality was assessed by the Agilent RNA 6000 Nano Assay Kit (Agilent Technologies, Santa Clara, CA, USA). RNA integrity index (RIN) values ranged from 9.5 to 9.9 for cultured cells and from 6.7 to 9.3 for frozen sections. cDNA was synthetized with the High-Capacity cDNA Reverse Transcription Kit with RNase Inhibitor (Applied Biosystems™, Thermo Fisher Scientific brand) using a modified thermal profile: 10 minutes at 25°C, 60 minutes at 42°C, 5 minutes at 85°C) in a 10 µL or 20 µL reaction volume, for CTCs and frozen tissue sections or cell pellets, respectively.

Quantitative real-time PCR

Quantitative PCR analysis was performed in technical triplicates using the maximum cDNA volumes allowed, for tumor cell indirect quantification or gene expression analysis in CTC subpopulations, or cDNA obtained from inputs of 10 or 30 ng of pre-quantified total RNA, for single gene expression analysis in cell line models, in TaqMan® Universal PCR Master Mix (Applied Biosystems™, Thermo Fisher Scientific), according to the standard thermal profile set on the 7900HT Fast or QuantStudio™ 12K Flex Real Time PCR System (Applied Biosystems™). Indirect quantification of tumor cells in xenograft samples was performed by interpolating the threshold cycle (C_t) values obtained by a TaqMan® assay (Applied Biosystems™) specific for

human *ACTB* (Hs03023943_g1; undetected in Mouse Universal Reference Total RNA from Clontech (Mountain View, CA, USA)) on a standard curve obtained from several amounts of MDA-MB-231 cells (2-5-10-20-40-100-200-1,000-5,000-10,000-50,000-100,000), which were lysed in triplicate in the AdnaTest Lysis Buffer after direct spike-in of single cells (from 2 to 200 cells) or serial dilution from cell suspensions (when ≥200 cells). Other single TaqMan® assays used for gene expression analysis were: *TFF1* (Hs00907239_m1), *TFF2* (Hs00193719_m1), *TFF3* (Hs00902278_m1; undetected in *Mus musculus* cDNA), *FADS3* (Hs00960377_m1), *GAPDH* (Hs00266705_g1), *RPL13A* (Hs04194366_g1). The C_t for tumor cell quantification by human *ACTB* assay was manually set to 0.2, while for single gene expression analysis the C_t was automatically set by the 7900 SDS v2.4 software (Applied Biosystems™). Analysis of relative gene expression was performed applying the $2^{-\Delta\Delta CT}$ method.

Low-density array gene expression profiling

Gene expression profiling of CTCs from clinical samples was performed by quantitative PCR in technical duplicate using custom 384-Well Microfluidic Card TaqMan® Gene Expression Assays (Applied Biosystems™). Target specific pre-amplification was performed using a Custom TaqMan® PreAmp Pool **(Supplementary Table S6)**, according to the manufacturer's instructions. PCR was run in technical duplicate using the 7900HT Fast Real-Time PCR System (Applied Biosystems™). Data analysis was performed using the Relative Quantification analysis module RQ Manager version 2.3. The relative threshold method (C_{rt}) was used to set the threshold cycle (equivalent C_q), which accounts for low reaction volumes and associated differences in fluorescence levels. Two quality control parameters were set before performing C_{rt} analysis: the AMP score (rejection threshold <1.0) and the Calculated confidence in the C_q value (rejection threshold <0.8), both related to the performance of the amplification, scored according to an ideal sigmoidal amplification curve. Mean equivalent C_q values obtained after C_{rt} were used for subsequent gene expression analyses. For CTC gene expression analysis, the positivity threshold for each target gene was set according to the mean equivalent C_q obtained from 5-mL blood samples of female healthy donors, processed by the AdnaTest® EMT-2/Stem CellSelect kit. For genes with C_q undetermined in blood samples of healthy donors, ΔC_q for each clinical blood sample was calculated as ΔC_q=[Cut-off(gene) − BloodSample C_q(gene)], while for genes expressed (i.e., C_q <40) in healthy donors (leukocyte contribution), ΔC_q for each clinical blood sample was calculated as ΔC_q=[Cut-off(gene) − BloodSample C_q(gene)] − [Cut-off(*PTPRC*) − BloodSample C_q(*PTPRC*)] [22], where each gene cut-off was calculated as a mean-2SD of C_q values obtained in healthy donors. For gene expression analysis in tumor cells obtained from fine-needle biopsies, ΔC_q=[C_q(*GAPDH*) – C_q(gene)]. To assess the CTC kinetics, we classified as cases with unfavorable CTC trend those who were CTC+ve before starting and during the course of therapy, whereas all the other CTC detection patterns were included in the class of cases with favorable CTC trend.

Microarray gene expression profiling

RNA samples extracted from the MDA-MB-231 xenograft model and from the parental cells (50 ng pre-quantified total RNA inputs or RNA volumes corresponding to 5,000 tumor cells according to the indirect quantification method) were profiled using the Illumina Human Whole-Genome DASL HT Assay (Illumina, Inc., San Diego, CA, USA), according to the manufacturer's

instructions. The assay sensitivity when analyzing low-input RNA samples were already demonstrated in a previous report [21]. Universal Human Reference RNA from Stratagene (San Diego, CA, USA) and Mouse Universal Reference Total RNA from Clontech (Mountain View, CA, USA) were used as positive and species-specificity technical controls, respectively. The BeadChips were imaged on the BeadArray Reader. Illumina BeadScan software was used for image acquisition.

Candidate gene selection criteria

The panel of 17 MDA-MB-231 model-derived genes whose expression was analyzed in clinical samples includes 13 genes up-regulated in CTCs compared to solid lesions in xenograft models when considering the list of gene expression data obtained from experiments GEP1 and GEP2 (listed in Supplementary File S1), and 4 genes found up-regulated in CTC compared to primary tumor and not differentially expressed in MDA-MB-231 parental cells line in experiment GEP1 (listed in Supplementary Table S2). *PTPRC* encodes the common leukocyte antigen CD45, and was used as control of cell capture by AdnaTest. The total number of genes for clinical studies was a priori established limited to 20 in order to minimize possible biases caused by probes cross-reactions during pre-amplification and low-density array analysis. Genes from the list of expression data from GEP1 and GEP2 were selected after excluding those whose biological role in metastasis or in some steps of the metastatic cascade was already known and those with counterintuitive role in metastasis. Genes from GEP1 data were selected after excluding those with known role in metastasis or tumor growth and focusing on some genes described in other models: the *TFF3* family, involved in several biological functions in other cancer models or breast cancer subtypes, and *ELF3*, reported in another breast CTC model.

For the development of the CTC-specific signature for clinical studies, we first assessed the detection of the selected 17 genes in 12 healthy donors, and then excluded those expressed in healthy donors or with negligible detection frequency in patients (i.e., *TFF2* and *TPPP*). The resulting 5-gene panel was the sequent: *ADPRHL1*, *ELF3*, *FCF1*, *TFF1* and *TFF3*.

ELISA assay

Conditioned medium was collected after 72 hours from seeding or transfection or selection under Puromycin, centrifuged at 1,000 RCF for 5 minutes at 4°C, and stored at -80°C. Secreted TFF3 concentrations were measured in technical duplicate or triplicate by the Human TFF3 Quantikine® ELISA Kit (R&D systems, Minneapolis, MN, USA), according to the manufacturer's instructions. The assay was verified by the manufacturer for not interfering with TFF1, TFF2 and other secreted proteins. TFF3 concentrations (pg/mL) were calculated using a standard curve.

Protein extraction and Western blotting

Protein lysates were obtained from frozen cell pellets using standard lysis buffer (50 mM TRIS-HCl pH 7.4, 250 mM NaCl, 5 mM EDTA, 1% Triton X-100) supplemented with 5mM NaF, 1 mM $Na_4P_2O_7$, 5 mM Glycerol-2-Phosphate, 1 mM 4-(2-aminoethyl)benzenesulfonyl fluoride hydrochloride, 10 µg/mL leupeptin, 1 µg/mL aprotinin, 1 µg/mL pepstatin A. HUVEC protein lysate was kindly provided by the Molecular Targeting Unit at INT. Protein quantification was performed by the Micro BCA™ Protein Assay kit (Pierce Biotechnology, Rockford, IL, USA),

following the manufacturer instructions. Protein extracts (20 µg) were denatured in NuPAGE® LDS non-reducing sample buffer and run in precast NuPAGE® Bis-TRIS Mini Gels at a 100 V constant voltage (Invitrogen, Thermo Fisher Scientific brand). Proteins were blotted by the XCell II™ Blot at a 30 V constant voltage (Invitrogen) and visualized by staining with Ponceau S solution (Sigma-Aldrich). Filter papers were incubated with anti-vinculin rabbit polyclonal antibody (catalog number ab155120, Abcam, Cambridge, UK) and anti-FADS3 mouse monoclonal antibody (catalog number H00003995-M07, clone 3D2, Novus Biologicals, Centennial, Colorado, USA) in 5% nonfat dried milk for 16 hours at 4°C. Protein signals were detected by the ECL Plus™ chemiluminescence reagent (GE Healthcare, Buckinghamshire, UK) following the manufacturer instructions.

Immunofluorescence and immunohistochemistry staining

Immunofluorescence was performed on cell lines cultured on poly-L-Lysine-coated coverslips (Sigma-Aldrich) and fixed for 8 minutes with 2% neutral buffered formalin, or cells filtered through the ScreenCell® Cyto kit and fixed on porous isolation supports (ISs) with the ScreenCell proprietary FC2 buffer. Samples were rehydrated in TBS pH 7.4 (Bio-Optica), permeabilized with 0.01% Triton-X100 – for the analysis of intracellular proteins – and blocked for non-specific binding with 5% bovine serum albumin (BSA, Sigma-Aldrich) in TBS for 30 minutes. Cells were stained over night at 4°C with antibodies diluted in 5% BSA-TBS: rabbit monoclonal Alexa Fluor® 488-conjugated antibody specific for human cytochrome c oxidase subunit IV (COX IV, catalog number 4853, clone 3E11, isotype IgG; Cell Signaling Technology, Danvers, MA, USA) diluted 1:100, no cross-reaction with murine tumor cells previously verified [18]; mouse monoclonal antibody anti-human EpCAM/TROP1 diluted 1:100 (catalog number NBP2-44636, clone EGP40/1120, Novus Biologicals) conjugated with Alexa Fluor® 647 or 594 by the Microscale Protein Labeling kit (Molecular Probes, Invitrogen™, Thermo Fisher Scientific brand), specific for the human protein and non-reacting with murine tumor cells; mouse monoclonal antibody anti-EpCAM/CD326 (catalog number MA5-12436, clone 323/A3, Pierce, Thermo Fisher Scientific brand), conjugated with Alexa Fluor® 488 by the Microscale Protein Labeling kit, which reacts both with human and murine EpCAM; rabbit monoclonal anti-human TFF3 antibody (catalog number ab109104, clone EPR3973, Abcam) diluted 1:100, conjugated with Alexa Fluor® 488 or 594 by the Microscale Protein Labeling kit antibody, which reacts both with human and murine; rabbit polyclonal anti-TFF3 antibody (catalog number ab101099, Abcam) diluted 1:160, conjugated with Alexa Fluor® 488 by the Microscale Protein Labeling kit, which does not react with murine TFF3. Nuclei were stained with 5 µg/mL 4',6-diamidino-2-phenylindole (DAPI) dilactate solution (Sigma-Aldrich). ISs were mounted with the Aqueous Fluoroshield Anti-Fade Fluorescence Mounting Medium (Abcam). Coverslips were mounted with Aqueous Fluoroshield Mounting medium with DAPI (Abcam). Cell count and image digitalization were performed by Nikon Eclipse TE2000-S fluorescence microscope with NIS-Elements imaging software. Single CTCs were defined as COX IV positive cells not in direct contact (i.e., juxtaposed membranes) with other COX IV positive cells, and CTC clusters were defined as aggregates of at least 2 COX IV positive cells, according to criteria for CTC identification previously described [18]. Multiplex immunofluorescence for the analysis of CTC subpopulations was performed on a cytospin obtained from blood samples of one MDA-MB-231

xenograft with about 200,000 CTCs. CTC subpopulation count was performed on 14 fields under a 40x objective.

For immunohistochemistry analysis, 10 and 2 formalin-fixed paraffin-embedded sections of lungs and lymph nodes, respectively, were cut from the distal and/or the proximal part of the organ core, stained using a monoclonal antibody against human COX IV (catalog number 4850, clone 3E11, Cell Signaling Technology). Antigen retrieval was performed at 95°C for 30 minutes in Sodium Citrate Buffer (10 mM Sodium Citrate, 0.05% Tween 20, pH 6.0). Samples were incubated with the primary antibody diluted 1:1,000 in Antibody Diluent (Dako), overnight at 4°C. Antibody visualization was obtained using the EnVision®+ System-HRP Labelled Polymer (Dako). Nuclei were counterstained with Mayer hematoxylin (Bio-Optica). Lung metastases (COX IV-positive cells) were digitalized under a 10x objective with a Nikon Eclipse E-600 optical microscope and quantified by densitometric analysis using the ImageJ software, version 1.51g (https://imagej.nih.gov/ij/). Lung metastasis extent was obtained by the sum of the signals measured per each COX IV+ve area in the first, fifth and tenth section.

Flow-cytometry analysis

For EpCAM analysis, MCF7 and MDA-MB-231 cells were harvested using EDTA 2 mM and suspensions of $5x10^5$ cells were washed in PBS-BSA 0.03% and incubated with mouse monoclonal anti-human CD326 (EpCAM) PerCP-Cy5.5-conjugated antibody (catalog number 347199, clone EBA-1, BD Biosciences) in 300 µl of PBS-BSA 0.03% for 30 minutes at 4°C; mouse IgG2b κ light chain (0.06 µg) was used for isotype control. Cells were then washed, diluted in 500 µL of DMEM/F-12 supplemented with 5% FBS and 2 mM EDTA, and analyzed by BD FACSAria™ II flowcytometer (Becton Dickinson, Franklin Lakes, NJ, USA) at an excitation wavelength of 488 nm and an emission wavelength of 677 nm, using FlowJo™ software (BD Biosciences).

Bioinformatic analysis

Raw data from microarray analysis were pre-processed using the R/Bioconductor package "lumi" (www.bioconductor.org). Samples that clustered separately from the three main clusters corresponding to the tissue sources named Disseminated cells, Parental cell line and Solid lesions, and with median $\log_2$(signal intensities) lower than those of the majority of samples, i.e., lower than 6 in gene expression profile experiment 1 or lower than 9 in gene expression profile experiment 2 (samples named 70X PTi (d) in GEP1, and 152X PTa (d), 147X LNa (c), and 147X LUNG (c) in GEP2), and Mouse Universal Reference Total RNA control samples, were excluded from normalization step and downstream analyses. Data were $\log_2$ transformed and normalized using the robust spline normalization method [23]. Only probes with a detection *p*-value<0.01 in at least one sample were retained, while those not associated to HUGO gene symbols (www.genenames.org) were filtered out. Multiple probes mapping to the same gene were collapsed selecting the probe with the highest inter-quartile range across samples. Differential expression analysis was performed using the "limma" package [24]. *P*-values obtained from limma were adjusted for multiple-testing using the Benjamini-Hochberg false discovery rate (FDR). Principal variance component analysis (PVCA) was performed using the "pvca" package to determine which sources of biological and technical variability are more prominent in a microarray dataset. Unsupervised hierarchical clustering was performed using the most variable

genes (interquartile range intensities >95th percentile) and average linkage and 1-Pearson's correlation as distance measure. Gene Ontology (GO) analysis was carried out on the list of genes found differentially expressed in GEP1 and validated in GEP2 using the "topGO" package. To summarize the results of GO analysis the overlap coefficient (OC) between each pair of significant GO terms was calculated as a measure of similarity, and terms with an OC ≥0.5 were visualized as a network using Cytoscape version 3.2.1 (https://cytoscape.org/) and manually annotated. Raw and normalized data are available at NCBI GEO (Gene Expression Omnibus) repository with accession number GSE188899.

Statistical analysis

For in vitro and in vivo experiments, a descriptive statistical analysis was performed by reporting means with Standard deviations (SD) or medians with Interquartile ranges (IQR) in the graphical representations of data. For continuous variables, group comparison was performed by resorting to non–parametric exact approaches (i.e., Wilcoxon's exact test). Mixed models (with a compound symmetry covariance matrix) were fitted to assess the tumor growth (on logarithmic scale) as a function of time and experimental group (fixed factors) with mice considered as random factor. The same approach was used to compare the optical density in time course cell proliferation assays.

Associations between categorical variables were tested by the Chi-square statistic or Fisher's exact test whenever appropriate. Event-free survival (EFS) was defined as time from blood collection before the administration of therapy to event, i.e., disease distant relapse or progression, respectively for M0 and M+ patients. The pattern of EFS according to the clinical setting was estimated using the Kaplan–Meier method, and the curves of early and advanced breast cancer patients were compared using the log-rank test. The role of the clinical setting in EFS was assessed using Cox regression. For the prognostic assessment of the considered CTC-based tests, Relapse-free survival (RFS) and Progression-free survival (PFS) were defined as time from blood collection to distant relapse or to progression, respectively for M0 and M+ patients. The patterns of RFS and PFS according to the considered variables were estimated using the Kaplan–Meier method, and the curves were compared using the log-rank test.

The role of the considered variables in RFS and PFS was assessed using the Firth's bias correction method for Cox regression models.

All statistical analyses were carried out with SAS (Statistical Analysis System, RRID:SCR_008567, version 9.4; SAS Institute, Inc., Cary, NC, USA), adopting an α level of 5%. Graphical representations were obtained with Prism version 9.2 (GraphPad Software, San Diego, CA, USA).

Supplementary Figures

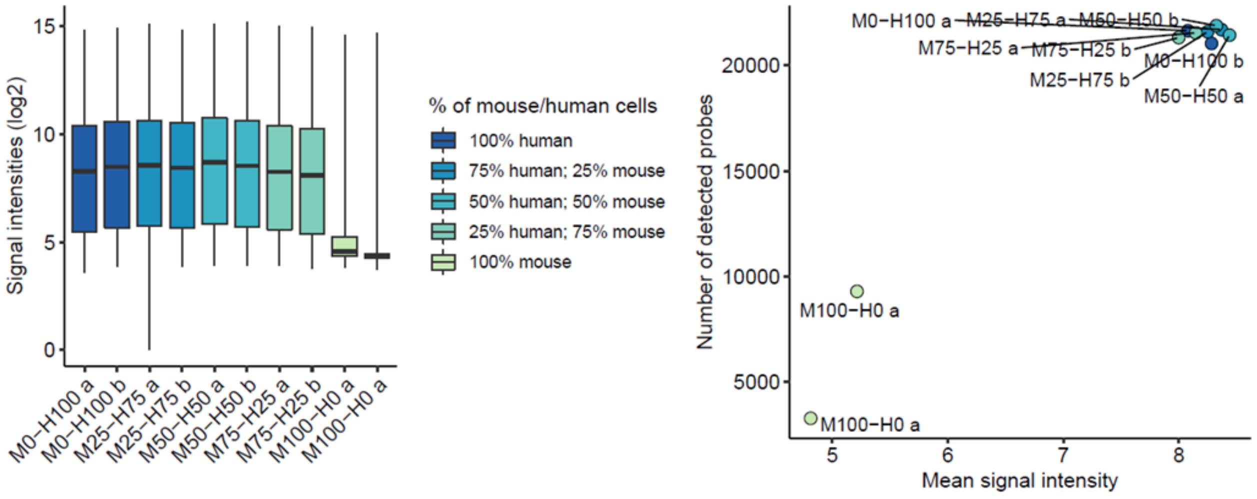

Supplementary Fig. S1. (left) Box and whiskers plot of log_2(intensities) and **(right)** scatter plot representation of mean signal intensities and detection rates using GEP data from human and murine universal RNA reference samples. Data were obtained from technical duplicates ("a" and "b") of human ("H") and murine ("M") universal RNA reference samples mixed at different ratios (numbers indicate the RNA percentage).

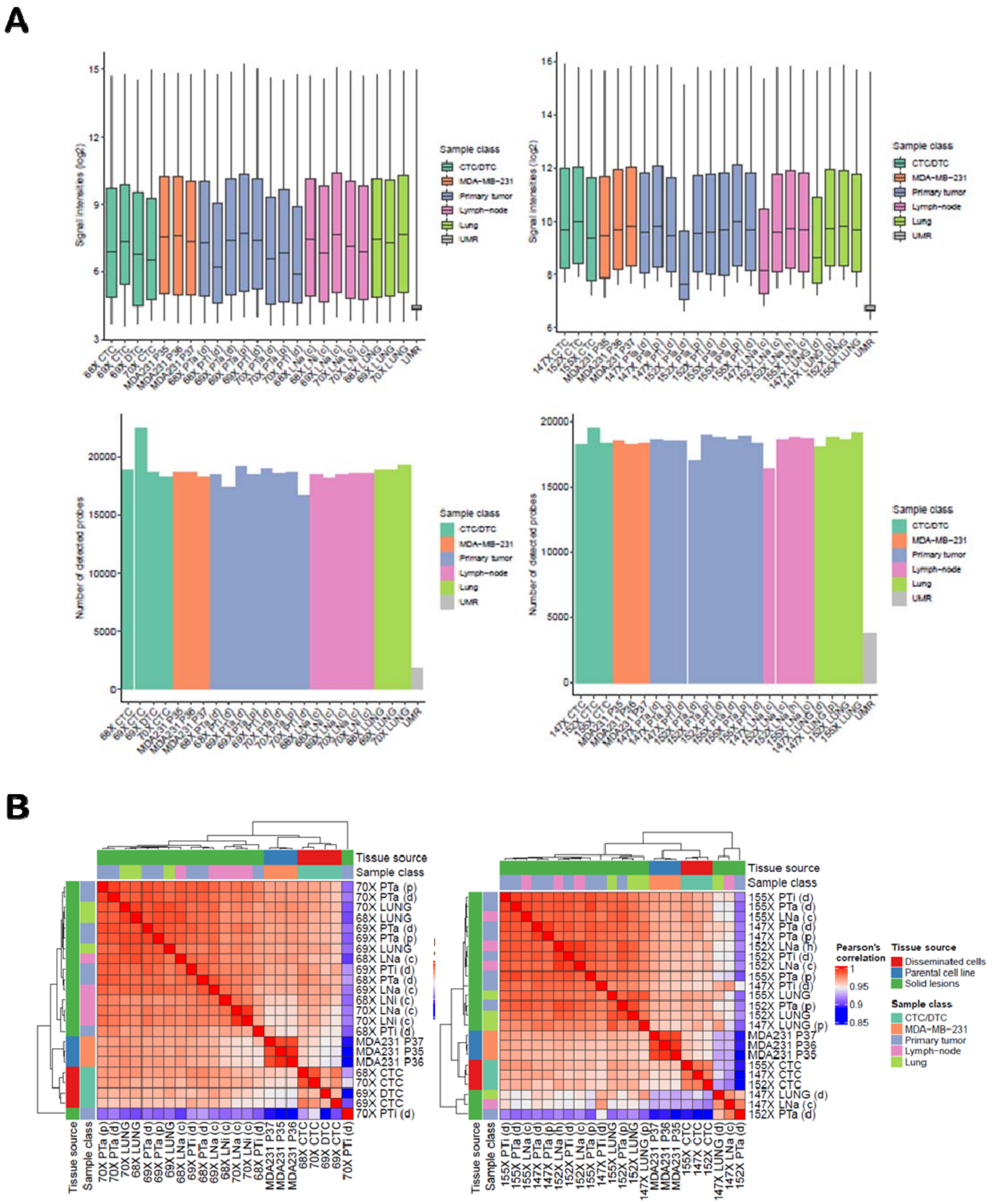

Supplementary Fig. S2. A, (top) Box and whiskers plots of the distribution of raw log_2(intensity) signals in **(left)** gene expression profile (GEP) GEP1 and **(right)** GEP2 experiments with MDA-MB-231 xenograft models, and **(bottom)** bar chart of the number of detected genes in **(left)** GEP1 and **(right)** GEP2 experiments. **B,** Heat map of pair-wise correlations of raw expression data in **(left)** GEP1 and **(right)** GEP2 experiments.

C

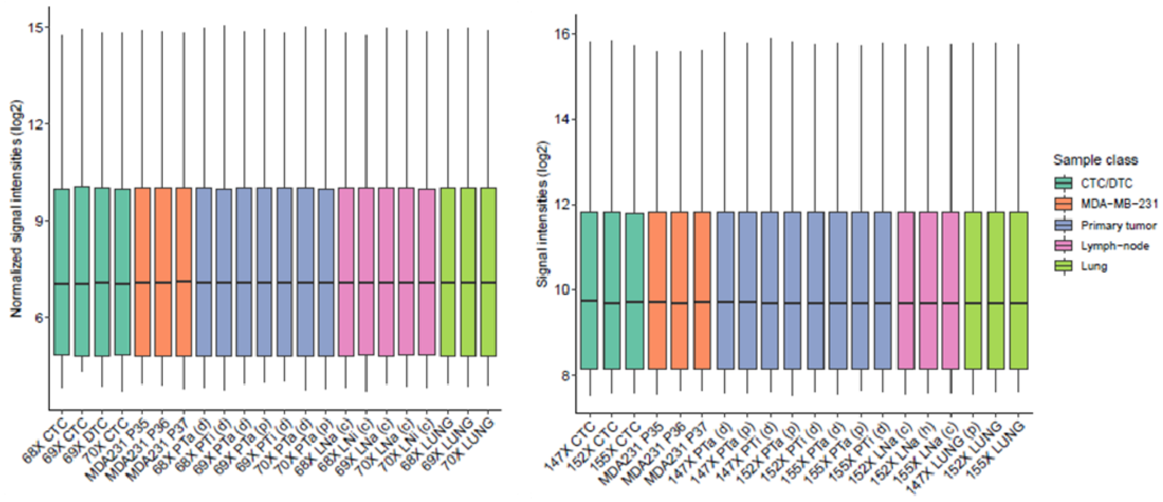

D

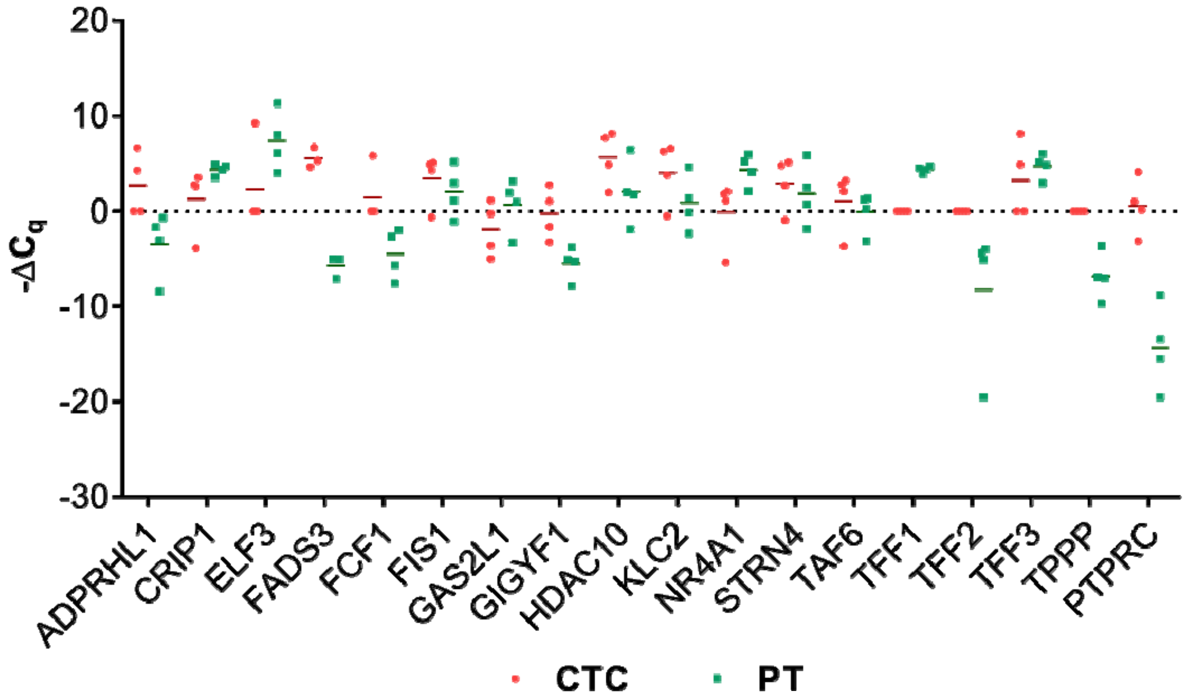

Supplementary Fig. S2. (continued) C, (top) Box and whiskers plots of the distribution of normalized $\log_2$(intensity) signals in **(left)** GEP1 and **(right)** GEP2 experiments. **D,** Dot plot of individual gene expression values in circulating tumor cell-enriched blood (CTC) and primary tumor fine-needle aspirate (PT) samples of breast cancer patients (N=4).
Abbreviations in the figure: “a” and “b”, technical replicates; “H”, human universal RNA reference; “M”, universal RNA reference; numbers indicate the RNA percentage; “CTC”, circulating tumor cells; “DTC”, tumor cells disseminated to bone marrow; “PT”, primary tumor; “LN”, lymph-nodal metastasis; “LUNG”, lung metastasis; “a”, axillary; “i”, inguinal; “(c)”, controlateral to PT; “(h)”, homolateral to PT; “(d)”, distal; “(p)”, proximal. MDA-MB-231 passage number in culture is indicated after “P”. Animal ID is indicated before “X”.

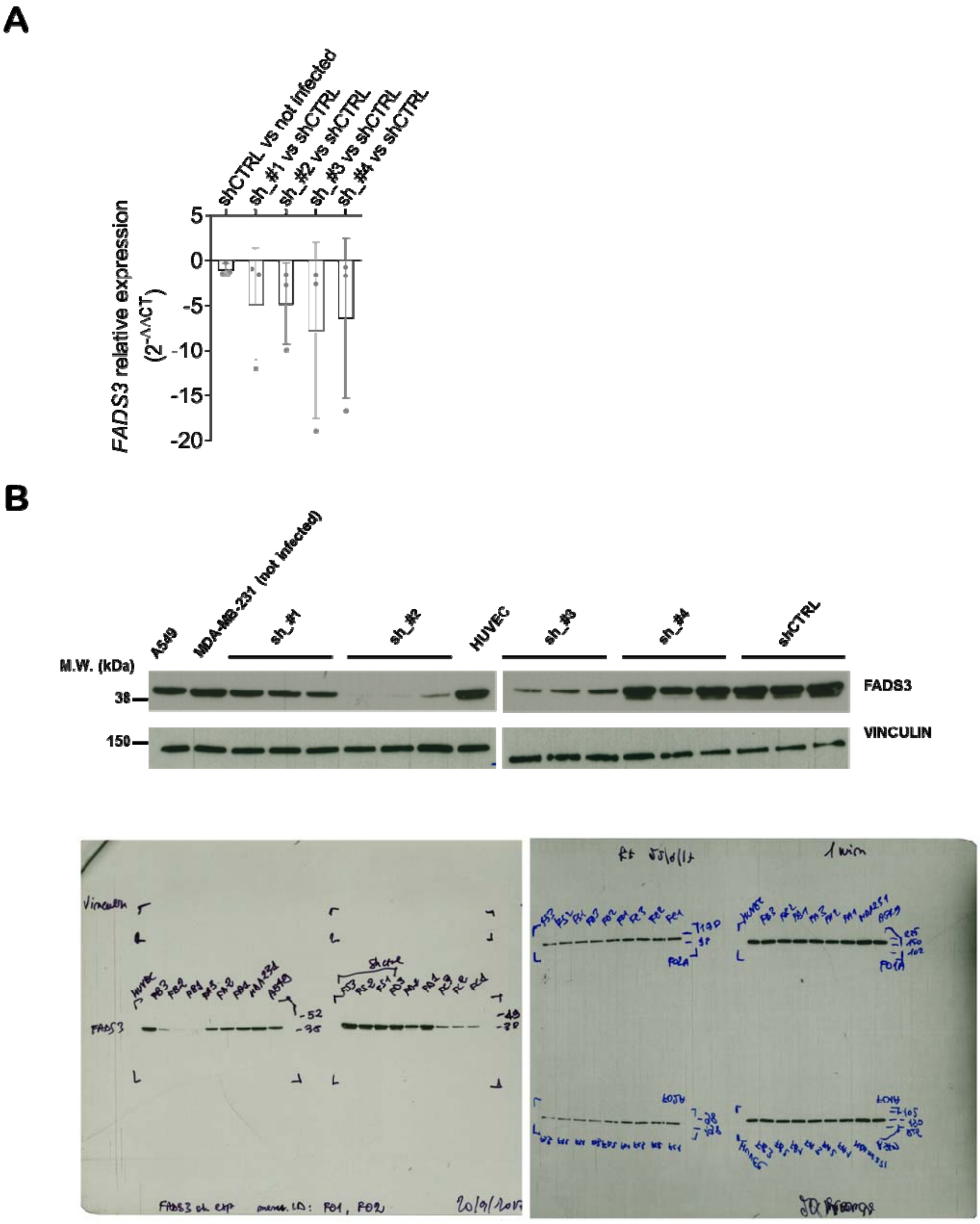

Supplementary Fig. S3. A, Bar chart of *FADS3* mean±SD expression fold-change ($2^{-\Delta\Delta Ct}$, *RPL13A* endogenous control) and **B,** FADS3 and vinculin chemiluminescence signals detected by Western blotting in MDA-MB-231 cells infected with three different shFADS3 lentiviral particles (indicated as “#1”, “#2”, “#3”) versus controls (N=3 lentiviral infection replicates).

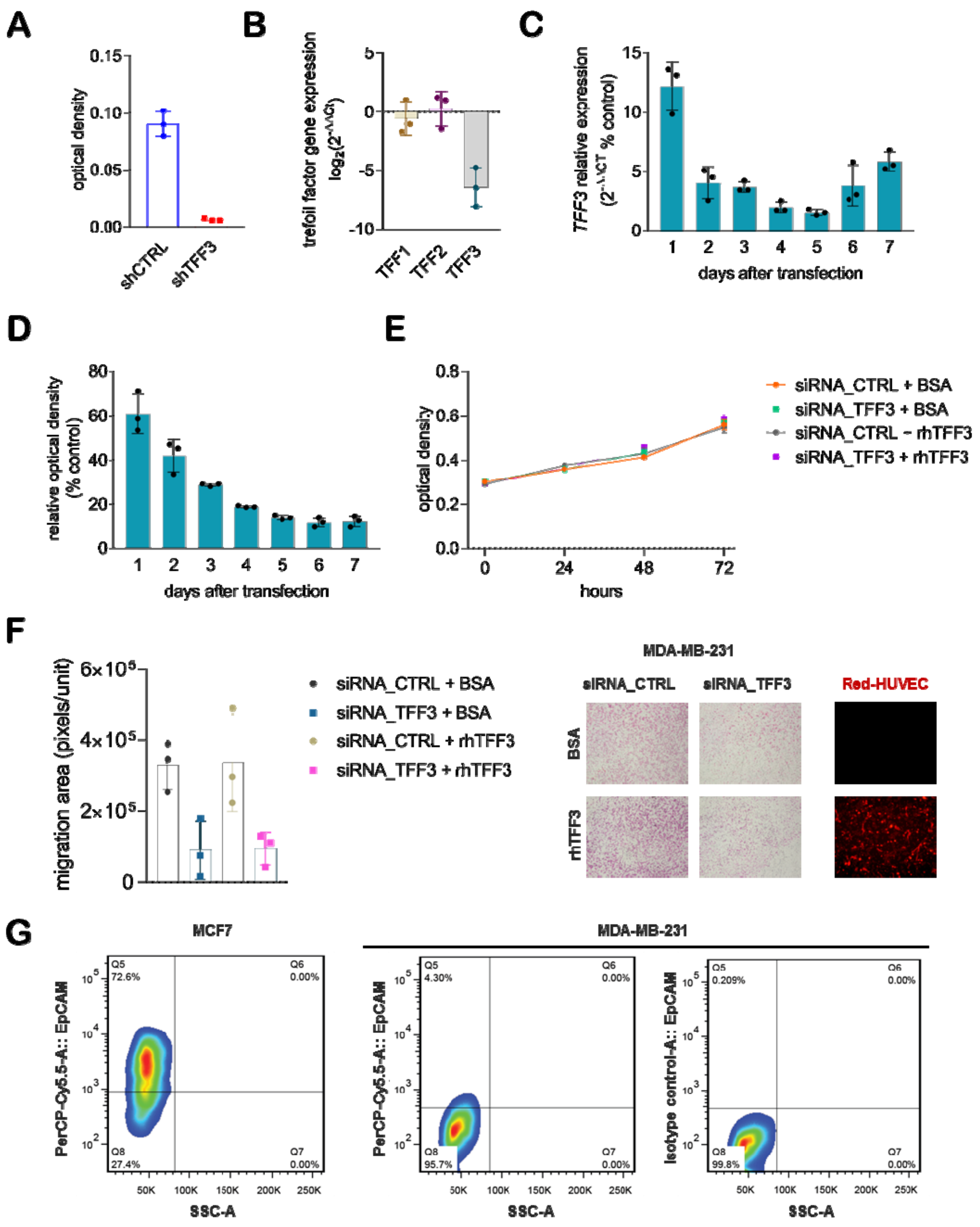

Supplementary Fig. S4. A, Dot plot of optical density measured in conditioned media upon TFF3 stable knock-down in MDA-MB-231 cells (N=3 lentiviral infection replicates). **B,** Dot plot of *TFF1*, *TFF2* and *TFF3* expression normalized to *GAPDH* upon TFF3 stable knock-down versus controls in MDA-MB-231 cells (N=3 lentiviral infection replicates). **C,** Dot plot of *TFF3* expression normalized to *RPL13A* upon *TFF3* transient silencing in MDA-MB-231 cells (N=3 transfection experiments). **D,** Dot plot of TFF3 relative concentration in conditioned media upon *TFF3* transient silencing versus controls in MDA-MB-231 cells (N=3 transfection experiments). **E,** Line chart representation of the mean±SD optical density measured in conditioned media in a

time-course proliferation assay upon *TFF3* transient silencing in MDA-MB-231 cells (N=3 observations in 3 transfection replicates). **F, (left)** Dot plot of MDA-MB-231 migration area (mean of 3 wells) measured by Boyden chamber assay (N=3 experiments) and **(right)** representative images of sulforhodamine B stained cells (4x objective) and red-fluorescent HUVEC cells as positive control (10x objective) at the chamber bottom side. **G,** Density plot of the percentage of surface EpCAM-PerCP-Cy5.5-positive events measured by flowcytometry. Abbreviations in the figure: BSA, bovine serum albumin; rhTFF3, recombinant human TFF3.

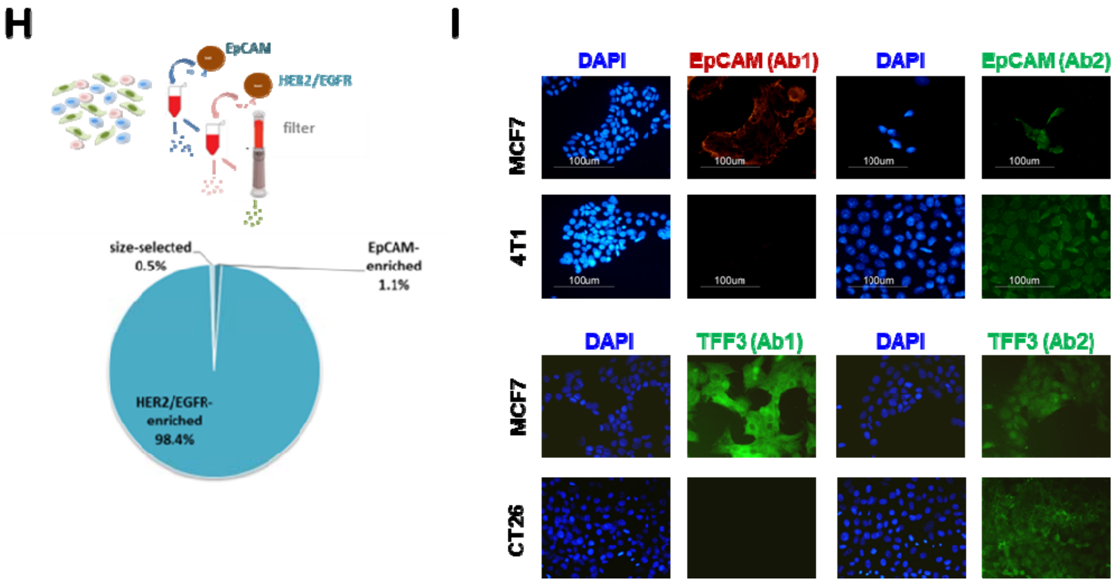

Supplementary Fig. S4. (continued) H, (left) Scheme of circulating tumor cell (CTC) sequential capture approach and **(right)** pie chart of each CTC subpopulation percentage in MDA-MB-231 xenograft models (N=6). **I,** Representative single-fluorescence channel images (40x objective) of human and murine tumor cells stained with anti-EpCAM or anti-TFF3 antibodies (conventionally indicated as “Ab1” or “Ab2”).

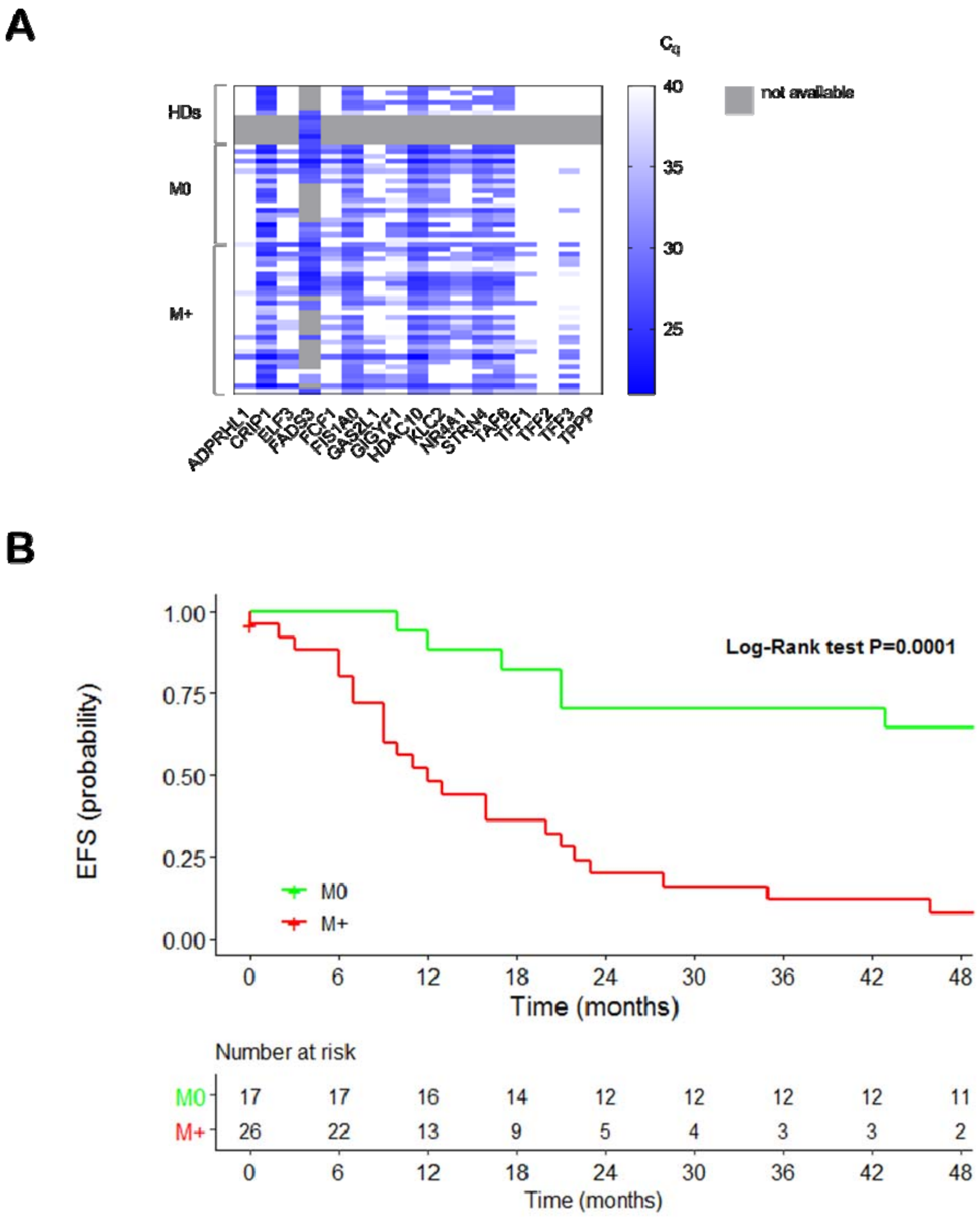

Supplementary Fig. S5. A, Heatmap representation of C_q values for a panel of genes up-regulated in MDA-MB-231-derived circulating tumor cells (CTCs) assessed in blood samples of healthy donors (“HDs”) and non-metastatic (“M0”) and stage IV (“M+”) breast cancer patients processed by AdnaTest CellSelect kits. **B,** Kaplan-Meier plot of four-year Event-free survival (EFS) probability according to the clinical setting (M0 and M+ breast cancer patients).

Supplementary Tables

**Supplementary Table S1. CTC load estimated by a PCR-based indirect quantification assay**						
GEP1 experiment				GEP2 experiment*		
Animal ID	CTC total number	CTC/mL	DTC total number	Animal ID	CTC total number	CTC/mL
63X	0	0	NA	**147X**	**9,137**	36,548
65X	3	3	0	148X	25	31
66X	30	100	0	149X	268	412
67X	0	0	NA	150X	821	1,173
**68X**	**21,811**	24,234	NA	151X	565	807
**69X**	**36,092**	72,184	17,884	**152X**	**15,057**	30,114
**70X**	**34,431**	57,385	54	154X	172	246
76X	1,714	3,116	NA	**155X**	**62,200**	69,111
77X	48	96	NA	156X	513	855
78X	172	191	262	157X	5,442	6,047

Abbreviations:
“CTC”, circulating tumor cell; “GEP” gene expression profile; “DTC”, tumor cells disseminated to bone-marrow; “NA”, not available.
*DTC isolation not performed
In bold: animals used for GEP experiments

Supplementary Table S2. List of genes obtained after multiple comparison analysis MDA-MB-231 versus (vs.) Primary Tumor (PT) and PT vs. Circulating Tumor Cells (CTC) and classified according to the expression level between CTC and MDA-MB-231

up/down	down/up	down/down	notDE/up	notDE/down
TFF2	*LST-3TM12*	*NCF1*	*C13ORF23*	*NR4A3*
TFF1	*SNCAIP*	*CRIP2*	*AOX1*	*LY6D*
NR4A1	*NAV3*	*C1QB*	*RN7SK*	*MIR196A2*
ELF3	*SNORD55*	*UBE2NL*	*SNORA45*	*PTGS1*
FLJ45445	*SNORA51*		*INO80D*	*CRYGB*
MMP28	*SNORA47*		*KIAA1632*	*BCAS1*
TFF3	*SNORD95*		*AGPAT5*	*CDH8*
ACSS1			*ZNF407*	*OR1L4*
INHBB			*JARID2*	*H19*
SPINK4			*SNORA53*	*SYN1*
IRX5			*KIAA2018*	*CITED1*
			SNORA25	*CDH29*
			SACS	*C6ORF27*
			CTNNB1	*RRAD*
			CRYBG3	*ADAMTS14*
			C2ORF86	*CD14*
			ZNF460	*MAMDC2*
			…	…

genes not differentially expressed (DE) in MDA-MB-231 vs. CTC

genes up-regulated in MDA-MB-231 vs. CTC

genes down-regulated in MDA-MB-231 vs. CTC

genes up- or down-regulated in CTC compared to both PT and MDA-MB-231

Supplementary Table S3. Association between CTC-specific genes and the clinico-pathological features in breast cancer patients																										
Variable		ADPRHL1+		ADPRHL1-		*p*-value	ELF3+		ELF3-		*p*-value	FCF1+		FCF1-		*p*-value	TFF1+		TFF1-		*p*-value	TFF3+		TFF3-		*p*-value
	Nev	N	%	N	%		N	%	N	%		N	%	N	%		N	%	N	%		N	%	N	%	
All patients	49	9	18.4	40	81.6		21	42.9	28	57.1		27	55.1	22	44.9		10	20.4	39	79.6		18	36.7	31	63.3	
Age (years)																										
<50	10	2	20.0	8	80.0	*1*[a]	6	60.0	4	40.0	*.2906*[a]	7	70.0	3	30.0	*.4778*[a]	1	10.0	9	90.0	*.6631*[a]	4	40.0	6	60.0	*1*[a]
≥50	39	7	17.9	32	82.1		15	38.5	24	61.5		20	51.3	19	48.7		9	23.1	30	76.9		14	35.9	25	64.1	
Clinical stage																										
M0	20	3	15.0	17	85.0	*.7199*[a]	6	30.0	14	70.0	*.1310*[b]	11	55.0	9	45.0	*.9905*[b]	0	0.0	20	100.0	*.0031*[a]	2	10.0	18	90.0	*.0013*[b]
M+	29	6	20.7	23	79.3		15	51.7	14	48.3		16	55.2	13	44.8		10	34.5	19	65.5		16	55.2	13	44.8	
ER and PgR status																										
Positive for either	42	6	14.3	36	85.7	*.1050*[a]	17	40.5	25	59.5	*.4427*[a]	23	54.8	19	45.2	*1*[a]	9	21.4	33	78.6	*1*[a]	16	38.1	26	61.9	*1*[a]
Negative for both	7	3	42.9	4	57.1		4	57.1	3	42.9		4	57.1	3	42.9		1	14.3	6	85.7		2	28.6	5	71.4	
HER2/*neu* status																										
Positive	9	3	33.3	6	66.7	*.3364*[a]	5	55.6	4	44.4	*.4698*[a]	6	66.7	3	33.3	*.4876*[a]	2	22.2	7	77.8	*1*[a]	3	33.3	6	66.7	*1*[a]
negative	40	6	15.0	34	85.0		16	40.0	24	60.0		21	52.5	19	47.5		8	20.0	32	80.0		15	37.5	25	62.5	
Subtype																										
Luminal	38	5	13.2	33	86.8	*.1513*[a] *.1786*[c]	15	39.5	23	60.5	*.6609*[a]	21	55.3	17	44.7	*.7649*[a]	8	21.1	30	78.9	*.6747*[a]	15	39.5	23	60.5	*.8594*[a] *.7238*[c]
HER2+	8	3	37.5	5	62.5		4	50.0	4	50.0		5	62.5	3	37.5		1	12.5	7	87.5		2	25.0	6	75.0	
Triple-negative	3	1	33.3	2	66.7		2	66.7	1	33.3		1	33.3	2	66.7		1	33.3	2	66.7		1	33.3	2	66.7	
Histotype																										
Ductal	33	6	18.2	27	81.8	*1*[a,d]	15	45.5	18	54.5	*.5368*[b,d]	22	66.7	11	33.3	*.0163*[b,d]	4	12.1	29	87.9	*.0455*[a,d]	10	30.3	23	69.7	*.0834*[b,d]
others	14	2	14.3	12	85.7		5	35.7	9	64.3		4	28.6	10	71.4		6	42.9	8	57.1		8	57.1	6	42.9	
Unknown	2	1	50.0	1	50.0		1	50.0	1	50.0		1	50.0	1	50.0		0	0.0	2	100.0		0	0.0	2	100.0	

[a] Fisher's exact test; [b] Chi-square test; [c] Luminal versus HER2+/Triple-negative [d] "unknown" were excluded from the test

Supplementary Table S3. Association between CTC-specific genes and the clinico-pathological features in breast cancer patients (continued)

Variable		ADPRHL1+		ADPRHL1-		*p*-value	ELF3+		ELF3-		*p*-value	FCF1+		FCF1-		*p*-value	TFF1+		TFF1-		*p*-value	TFF3+		TFF3-		*p*-value
	N_{ev}	N	%	N	%		N	%	N	%		N	%	N	%		N	%	N	%		N	%	N	%	
Tumor grade																										
G1 or G2	22	6	27.3	16	72.7	*.2431*[a,d]	9	40.9	13	59.1	*.5544*[b,d]	12	54.5	10	45.5	*.4906*[b,d]	4	18.2	18	81.8	*1*[a,d]	10	45.5	12	54.5	*.4906*[b,d]
G3	20	2	10.0	18	90.0		10	50.0	10	50.0		13	65.0	7	35.0		4	20.0	16	80.0		7	35.0	13	65.0	
Unknown	7	1	14.3	6	85.7		2	28.6	5	71.4		2	28.6	5	71.4		2	28.6	5	71.4		1	14.3	6	85.7	
Ki67																										
<10 %	5	0	0.0	5	100.0	*.5563*[a,d]	3	60.0	2	40.0	*.3600*[a,d]	2	40.0	3	60.0	*1*[a,d]	3	60.0	2	40.0	*.0313*[a,d]	2	40.0	3	60.0	*.6269*[a,d]
≥10%	28	6	21.4	22	78.6		10	35.7	18	64.3		14	50.0	14	50.0		3	10.7	25	89.3		8	28.6	20	71.4	
Unknown	16	3	18.8	13	81.3		8	50.0	8	50.0		11	68.8	5	31.3		4	25.0	12	75.0		8	50.0	8	50.0	

N_{ev}: number of evaluable cases

[a] Fisher's exact test; [b] Chi-square test; [c] Luminal versus HER2+/Triple-negative [d] "unknown" were excluded from the test

Supplementary Table S4. Univariate penalized Cox-regression analysis according to CTC status or CTC trend assessed by AdnaTest or CTC-specific signature in breast cancer patients

		M0[a]						M+[b]					
**Variable**	**Categories**	**N cases**	**N events**	**HR**	**95% CI**	**RFS (%)***	**95% CI**	**N cases**	**N events**	**HR**	**95% CI**	**PFS (%)***	**95% CI**
AdnaTest	positive	4	1	0.55	0.08-3.87	75	13-96	12	10	0.98	0.41-2.34	9	1-33
Reference	negative	10	5			50	18-75	12	11			25	6-50
AdnaTest kinetics	unfavorable	1	1	4.16	0.49-35.33	0	-	3	3	2.27	0.57-8.97	0	-
Reference	favorable	11	4			64	30-85	10	9			10	1-36
5-gene panel	positive	11	6	2.01	0.42-9.52	45	17-71	21	19	1.83	0.63-5.32	10	2-27
Reference	negative	6	2			83	27-97	5	4			40	5-75
5-gene panel_kinetics	unfavorable	6	5	4.67	1.06-20.61	17	1-52	10	10	1.82	0.57-5.79	0	-
Reference	favorable	10	3			80	41-95	5	4			20	1-58

CTC: Circulating Tumor Cells; M0: early stage breast cancer patients; M+: metastatic breast cancer patients HR: Hazard Ratio; CI: Confidence Interval; RFS: Relapse-Free Survival; PFS: Progression-Free Survival

[a] cases lost at follow-up (N=1) or treated with adjuvant therapy (N=2) were excluded from survival analysis

[b] cases lost at follow-up (N=3) were excluded from survival analysis

*at median follow up in the specific setting (87 and 32 months for M0 and M+, respectively)

Unfavorable: positive-positive; Favorable: negative-negative, positive-negative, negative-positive

**Supplementary Table S5. Number of animals and biological samples per experimental group for CTC and metastasis assays**					
Gene name	Experimental group	N° injected mice	N° tumor takes (2 mammary fat pads)	N° evaluable lung and lymph-node FFPE samples for metastasis analysis	N° evaluable cytological samples for CTC analysis
FADS3	Control group	8	16	6	8
	Gene knock-down group	7	14	7	7
TFF3	Control group	18	36	14	11
	Gene knock-down group	18	34	15	11
CTC: circulating tumor cell<br>FFPE: formalin-fixed paraffin embedded					

**Supplementary Table S6. List of genes and relative TaqMan® assay ID for low-density array gene profiling.**	
Target gene	Assay ID
ADPRHL1	Hs00293405_m1
CRIP1	Hs00832816_g1
ELF3	Hs00963881_m1
FCF1	Hs01056861_gH
FIS1	Hs00211420_m1
GAPDH	Hs02758991_g1
GAS2L1	Hs00977983_g1
GIGYF1	Hs01119153_g1
HDAC10	Hs00368899_m1
KLC2	Hs03988192_m1
NR4A1	Hs00374226_m1
PTPRC	Hs04189704_m1
STRN4	Hs01026676_m1
TAF6	Hs00425763_m1
TFF1	Hs00907239_m1
TFF2	Hs00193719_m1
TFF3	Hs00902278_m1
TPPP	Hs00389316_m1
